# Impact of a multifaceted intervention on physicians’ knowledge, attitudes and practices in relation to pharmaceutical incentivisation: protocol for a randomised control trial

**DOI:** 10.1136/bmjopen-2022-067233

**Published:** 2022-11-04

**Authors:** Muhammad Naveed Noor, Mishal Khan, Afifah Rahman-Shepherd, Amna Rehana Siddiqui, Sabeen Sharif Khan, Iqbal Azam, Sadia Shakoor, Rumina Hasan

**Affiliations:** 1 Department of Pathology and Laboratory Medicine, The Aga Khan University, Karachi, Sindh, Pakistan; 2 Department of Global Health & Development, London School of Hygiene and Tropical Medicine, London, UK; 3 Community Health Sciences, Aga Khan University, Karachi, Sindh, Pakistan; 4 Faculty of Infectious and Tropical Disease, The London School of Hygiene and Tropical Medicine, London, UK

**Keywords:** Protocols & guidelines, Health policy, MEDICAL ETHICS, EDUCATION & TRAINING (see Medical Education & Training), Quality in health care, Public health

## Abstract

**Introduction:**

In settings where the private sector constitutes a larger part of the health system, profit-gathering can take primacy over patients’ well-being. In their interactions with pharmaceutical companies, private general practitioners (GPs) can experience the conflict of interest (COI), a situation whereby the impartiality of GPs’ professional decision making may be influenced by secondary interests such as financial gains from prescribing specific pharmaceutical brands.

**Methods and analysis:**

This study is a randomised controlled trial to assess the impact of a multifaceted intervention on GPs’ medical practice. The study sample consists of 419 registered GPs who own/work in private clinics and will be randomly assigned to intervention and control groups. The intervention group GPs will be exposed to emotive and educational seminars on medical ethics, whereas control group GPs will be given seminars on general medical topics. The primary outcome measure will be GPs’ prescribing practices, whereas the secondary outcome measures will be their knowledge and attitudes regarding COI that arises from pharmaceutical incentivisation. In addition to a novel standardised pharmaceutical representatives (SPSR) method, in which field researchers will simulate pharmaceutical marketing with GPs, presurvey and postsurvey, and qualitative interviewing will be performed to collect data on GPs’ knowledge, attitudes and practices in relation to COI linked with pharmaceutical incentives. Univariate and multivariate statistical analyses will be performed to measure a change in GPs’ knowledge, attitudes and practices, while qualitative analysis will add to our understanding of the quantitative SPSR data.

**Ethics and dissemination:**

Ethics approval has been obtained from the Pakistan National Bioethics Committee (# 4-87/NBC-582/21/1364), the Aga Khan University (# 2020-4759-1129) and the London School of Hygiene and Tropical Medicine (# 26506). We will release results within 6–9 months of the study’s completion.

**Trial registration number:**

ISRCTN12294839.

**STRENGTHS AND LIMITATIONS OF THIS STUDY**

- The study assesses whether emotive sensitisation can help reduce profit-led prescribing.
- It evaluates the potential usability of the standardised pharmaceutical representatives method to ensure quality control.
- It uses a mixed-method approach to perform a comprehensive assessment.
- The inclusion of more female general practitioners would have provided additional insights.

## Introduction

The pharmaceutical industry is often a vital source of information for general practitioners (GPs) to learn about pharmaceutical products. This information helps GPs make informed choices and decisions in relation to patients’ diverse health needs.^1^ Nevertheless, the relationship between the pharmaceutical industry and GPs can negatively affect patients’ health and well-being, if/when incentives are mobilised to maximise profits through inappropriate prescribing.^2^ As a result of pharmaceutical incentivisation, GPs may experience a conflict of interest (COI)—a situation where GPs’ professional judgement is influenced by a secondary interest, such as financial gain, leading to a decision that conflicts with their interpretation of the patient’s best interest.^3^ There is evidence that GPs who engage in pharmaceutical incentivisation are likely to prescribe unnecessary and/or more expensive medicines to patients, further contributing to an increased financial burden on them.^4 5^ In addition, pharmaceutical incentivisation has important implications for antimicrobial resistance, as to achieve pharmaceutical targets, GPs may prescribe antibiotics to patients with common self-resolving ailments like cold and influenza.^6^

In Pakistan, the health system is composed of public and private sectors where a large proportion of patients access healthcare from private GPs.^7^ At the same time, the country is home to over 600 pharmaceutical companies including multinational, national and franchise-based local companies.^8^ To compete in the market, pharmaceutical companies use incentivisation as a major tool to maximise profits from GPs’ prescriptions.^9^ On the other hand, in Pakistan, poverty represents one of the key problems that people and the inextricable link between poverty and a lack of education and health awareness gives perfect grounds to the pharmaceutical industry and GPs to capitalise on patients’ weaknesses to maximise profits from pharmaceutical products, further exacerbating problems for patients.^10 11^

Our previous research in Pakistan helped us identify three interlinked health system constraints that shape private GPs’ decisions on receiving personal benefits from prescribing antibiotics.^12–16^ First, insufficient income generation from patient consultations alone—as patients are unwilling to spend substantial sums on medical advice without a prescription—resulted in a reliance on other sources of income.^15^ Second, limited opportunities for continuing medical education (CME) other than from pharmaceutical companies may lead to a close relationship with pharmaceutical sales representatives (PSRs).^13^ Third, inadequate access to diagnostic tools to establish the clinical need for antibiotics, making it easier for doctors to justify defensive overprescribing.^13^ We also found that private doctors engage in activities such as generating extra income from receiving monetary or non-monetary incentives, and sponsorships to attend academic events from pharmaceutical companies in return for meeting sales targets for specific medicines.^13^ These behaviours of private physicians stem from deeply embedded dynamics of pluralistic health systems. Hence, not only did these studies provide rich insights into critical health system and policy dynamics, but also helped to identify the issue of COI in medical practice and how this contributes to unnecessary prescribing of medicines in primary care settings.

Therefore, to improve the quality of primary care, actions are required that address pharmaceutical incentivisation.^17 18^ However, major impediments to progress on this front include limited assessment of interventions for improving the quality of care delivered by private GPs,^19 20^ and a marked absence of attention by researchers and policy makers as to the role of political, social, economic and cultural factors that influence the effectiveness and scalability of interventions.^21^ Furthermore, prohibiting or constraining private physicians through the enforcement of regulations may fail because of a lack of political and social support and enforcement capacity, as well as insufficient public sector capacity to provide care.^19^ Training interventions focusing on increasing technical knowledge and skills are the most common approach used to improve the quality of care.^22 23^ Our previous research also suggests that an intervention that can enhance GPs’ understanding of COI in medical practice might be useful to enable them to recognise potential actions that threaten professional ethics in their medical practice while interacting with PSRs.^13 15^ It is clear, however, from consistent evidence on the ‘know-do’ gap that COI related to profit generation from medicine sales plays a critical role in prescribing decisions, as do values associated with professional ethics and altruism.^24 25^ To improve GPs’ understanding of COI in medical practice, we aim to develop an intervention based on emotive and educational seminars and to test their impact on private GPs’ knowledge, attitudes and practices in relation to professional ethics in medical practice.

### Study objectives

The overarching aim of our study is to assess the impact of a multifaceted intervention for improving GPs’ prescribing practices in relation to COI linked with pharmaceutical incentivisation. The specific objectives of the study are to:

- Alter GPs’ prescribing practice in response to pharmaceutical incentives.
- Improve their knowledge of professional ethics in medical practice.
- Shift their attitudes to give primacy to patients’ well-being rather than pharmaceutical incentives.

## Methods

We plan to complete the study in three phases: formative, intervention and evaluation. The study is being undertaken in Karachi, which is the largest city by geographical area and population size in Pakistan.^26^ In 2021, we completed the formative phase of the study, whereas the intervention and evaluation phases will be completed in 2022.

### Formative work

We used semistructured interviews to obtain information from GPs, policy actors and PSRs about factors that lead to COI arising from pharmaceutical incentivisation. Individuals from regulatory institutions, professional medical associations and health communication/media consultants were deemed policy actors. One of the key objectives of the formative work was to codevelop a multifaceted intervention that could improve GPs’ knowledge, attitude and practices about professional ethics in medical practice. Preliminary analysis of our formative work suggests that in Pakistan professional ethics in medical practice have gradually changed and engaging in pharmaceutical incentivisation has become a norm. Competition in the pharmaceutical market, GPs’ desire to maximise income, combined with weaknesses in health regulation produce a context where an unethical relationship between the pharmaceutical industry and GPs is developed and sustained. Based on our findings, recommendations from the study participants and existing research, a multifaceted intervention has been developed in which, GPs have been invited to seminars to get exposure to messages on medical ethics.

### Intervention

**Figure 1:**
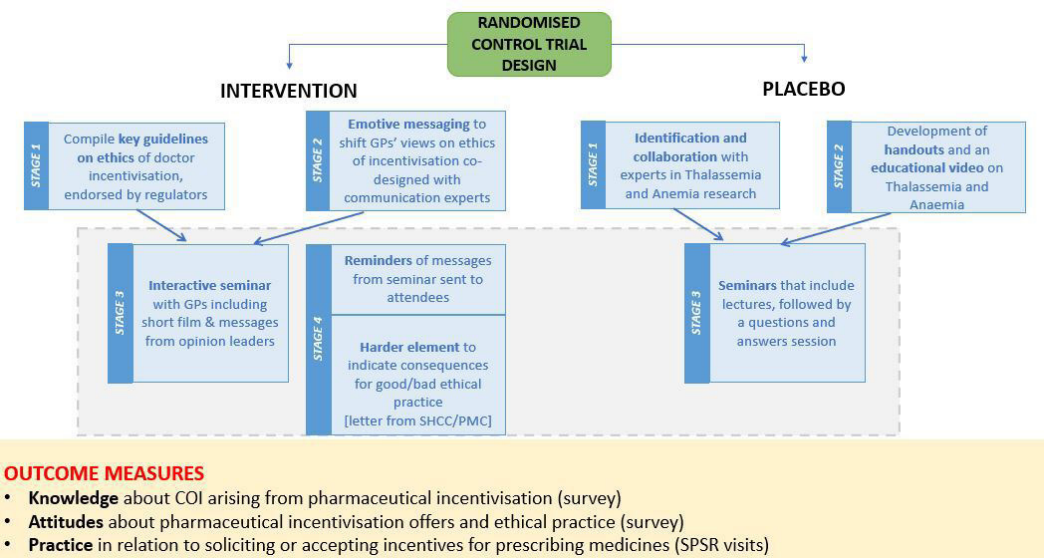
Diagram of the randomised control trial design. COI, conflict of interest; GP, general practitioner; PMC, Pakistan Medical Commission; ShcC, Sindh Healthcare Commission; SPSR, standardised pharmaceutical sales representative.

Our intervention is based on multifaceted seminars. As indicated in figure 1, the intervention group has been given seminars on ethics in relation to COI linked with pharmaceutical incentivisation, while the control group has received seminars on clinical issues such as thalassemia and anaemia. The intervention seminars have been developed in four major phases. First, international and national guidelines have been reviewed to develop a brief document on professional ethics in medical practice. The document covers several issues, including pharmaceutical incentivisation in the form of money, other material/nonmaterial benefits and the misuse of the medical license. The document has been endorsed by regulatory bodies at the federal and provincial levels, including the Pakistan Medical Commission (PMC) and the Sindh Healthcare Commission (SHCC). Second, in collaboration with media/communication experts, a filmmaker has been hired to produce an emotive video highlighting the effects of profit-led prescribing on patients’ well-being. Third, GPs have been invited to attend seminars on professional ethics in medical practice and on managing anaemia and thalassaemia in primary care settings. The intervention group GPs has also been sent the PMC-endorsed and SHCC-endorsed brief on ethics in medical practice (reinforcement) and the shortened version of the emotive video (reminder).

The control seminars have been developed in three phases. First, we identify experts in thalassemia and anaemia research and take them on board to teach these topics to the control group GPs. Second, in collaboration with experts, we develop a short educational video on thalassemia and handouts on the management of thalassaemia and anaemia in primary care settings. Third, we invite the control group GPs to attend the seminars.

### Outcome measures

The primary outcome measure of the study is the number of GPs who refuse to take unethical pharmaceutical incentives from the standardised PSRs (SPSRs). The secondary outcomes include GP’s change in knowledge and attitudes about professional ethics in medical practice.

### Participants

Private GPs, registered with the PMC and the SHCC, are deemed eligible to participate in the study. GPs outside Karachi, those working in welfare clinics, dentists, consultants and unqualified healthcare providers, are excluded from the study.

### Sample size

**Figure 2:**
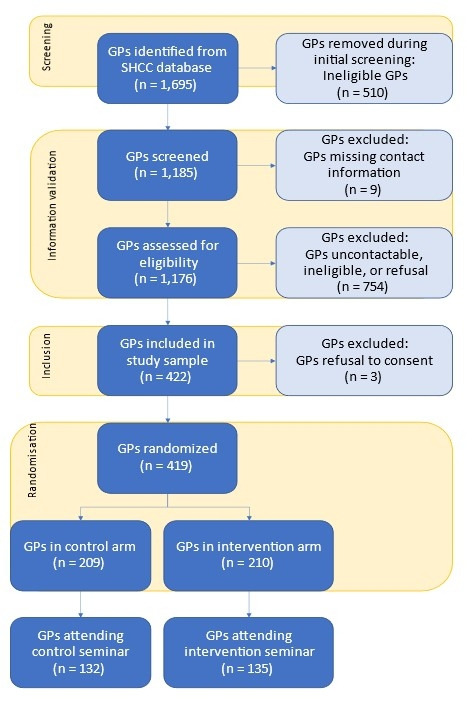
Flow chart of the sampling and randomisation process. GP, general practitioner; SHCC, Sindh Healthcare Commission.

For this study, a list of 1695 GPs, obtained from the SHCC, has been considered a sample universe. From it, 419 GPs have been sampled using a systematic sampling technique. In our literature review, we identify no relevant surveys of private GPs in Pakistan, so we use data from two studies on medical students and trainee doctors, to calculate the sample size for our study. One survey of medical students shows that 81% favoured pharmaceutical sponsorship of events at medical colleges^27^ and the other study on trainee doctors in public hospitals indicates that 57% did not know any code of medical ethics.^28^ We estimate that 80% of private GPs in our sample will be classified as ‘supporting pharmaceutical incentivisation’ and assume that our intervention shifts this to 65%, in line with changes observed in the few interventional studies addressing COI in medical professionals.^29 30^ Using these assumptions, a sample size of between 130 and 135 per group gives a power of 80% to detect a difference owing to the intervention. As indicated in figure 2, the sample of 419 GPs was extracted in three steps. First, the entire list is screened to exclude 510 healthcare providers who do not meet our eligibility criteria. Second, information validation is performed by calling the remaining 1185 healthcare providers to verify important information such as clinic addresses, registration status, level of education and whether they are active/inactive in medical practice, further leading to the exclusion of 764 names. As part of a baseline survey on GPs’ knowledge and attitudes about ethics in medical practice, three GPs have been further excluded from the sample who refuse to participate in the study.

### Randomisation

A total of 419 GPs, who completed the baseline survey and consented to attend the seminars, have been assigned to control and intervention groups, using a simple randomisation technique. Each GP in the list has been first assigned a unique identification number, following the sorting of the list with respect to six districts in Karachi, namely, East, West, Central, South, Korangi and Malir. Each GP has then been assigned a random number using the excel spreadsheet. Third, within each district, GPs are assigned to control and intervention groups by sorting the list. The intervention and control groups comprise of 210 and 209 GPs, of which, 135 and 132 GPs have attended the seminars. To prevent bias, we have collaborated with a statistician outside the research team to perform the randomisation process. Participants and data collectors are blinded in relation to GPs’ allocation to control and intervention groups.

### Data collection

To measure the impact of the intervention, our data collection is underway, and we anticipate completing it in September/October 2022. We adopt a multimethod approach to collect data on GPs’ knowledge, attitudes and practices in relation to COI linked with pharmaceutical incentivisation. Preintervention and postintervention surveys will be used to assess a change in GPs' knowledge of professional medical ethics and attitudes about engaging in pharmaceutical incentivisation. To collect information about their practices with respect to taking incentives for prescribing, a novel SPSR method will be used, in which trained field researchers will visit the GP clinics as pharmaceutical marketers to assess GPs’ reactions to pharmaceutical incentivisation offers. The SPSRs will recite a script (prepared by the research team) to introduce themselves and the imaginary pharmaceutical company and will market antibiotics commonly prescribed. In this process, the SPSRs make a clear incentivisation offer to GPs for prescribing the medicines and assess whether GPs accept/reject the offer. Since the SPSRs will be representing new pharmaceutical companies and there will be less familiarity between them and GPs, it is anticipated that there can be GPs’ responses, difficult to classify in terms of acceptance or rejection. Therefore, situations in which GPs ignore the SPSR offers or ask them to come back later will be classified as ‘undetermined’. The SPSRs will complete an electronic questionnaire after each GP visit to record information such as GPs’ demographic and clinical characteristics, and their responses to the incentivisation offer. To ensure the data quality, the research team will put several measures in place: recruiting field supervisors who will accompany the SPSRs to GP clinics and ensure the SPSRs’ responses in the questionnaire reflect the interactions with GPs, WhatsApp voice notes to the research team to summarise the interactions and monitoring the SPSR data considering the voice notes as a reference. The questionnaire will be analysed by research team members (also blinded to the allocation of GPs), who will assign a binary outcome for each GP such as ‘refused incentives immediately’, ‘refused incentives after being offered’, ‘asked for and accepted an incentive’, ‘offered and accepted an incentive’, ‘undetermined/undecided’. To prevent the SPSR assessment from being discovered as a simulation that not only can risk the intervention assessment but the SPSRs, in terms of their physical safety, each GP is paid a maximum of two visits. It is also important to note that the SPSRs are blinded to the allocation of GPs.

GPs have been made aware, through the consent procedure (online supplemental file 1) in the baseline survey, that their behaviour will be assessed covertly at some stage over 6 months but will not know details of the assessment to avoid the Hawthorne effect. We considered this novel SPSR approach to be superior to a revealed preference approach (eg, based on medical records) because records of prescribing by private physicians usually do not exist or are of poor quality in Pakistan, and to a stated preference design as it would rely on honest reporting of behaviour that is highlighted as unethical in the COI presentation.

10.1136/bmjopen-2022-067233.supp1 Supplementary data

Finally, to explain the undecided/undetermined SPSR assessment outcomes, we will conduct 20–25 qualitative interviews with GPs and get information on the reasons why some GPs ignore talking to PSRs, asking them to come back later, and what it means when a GPs uses terms like ‘I am saturated/packed/engaged’.

### Data analysis

The primary outcome will be assessed by comparing the proportion of GPs classified as ‘yes’ to accepting an unethical benefit (according to the regulator’s guidelines) for prescribing the SPSR’s antibiotic between the control and intervention groups. The proportions will be compared using a two-proportion z-test. Secondary outcomes, such as knowledge and attitude shifts, will be assessed by comparing mean scores in survey responses of control and intervention groups using unpaired t-test and proportions scores in survey responses using the z-test.

### Ethics and dissemination

Ethics approval has been obtained from the Pakistan National Bioethics Committee (# 4–87/NBC-582/21/1364), the Aga Khan University (# 2020-4759-1129) and the London School of Hygiene and Tropical Medicine (# 26506). Key ethical guidelines about informed consent, voluntary participation and maintaining participants' confidentiality will be observed throughout the research process.

Study results will be disseminated through open-access peer-reviewed publications targeting health promotion professionals, policy makers and researchers. The study will also be presented at academic conferences in medicine and public health.

### Patient and public involvement

None.

### Discussion

Our study is the first to evaluate the impact of a multifaceted intervention on GPs’ knowledge, attitudes and practices in relation to COI linked with pharmaceutical incentives: an area that has not been well studied in Pakistan. It may guide the design of further studies and interventions to improve prescribing practices in private primary care settings. We have attempted to develop a comprehensive approach to analyse the issue of pharmaceutical incentivisation and to suggest potential ways to address it. Our main objective is to examine the relationship between the pharmaceutical industry and GPs and to develop and test an emotive seminar-based intervention to improve GPs’ knowledge, attitudes and practices about professional ethics in medical practice.

The strengths of the study include the fact that it systematically uses different levels of investigation including formative work to understand how GPs, policy actors and PSRs conceptualise COI in medical practice, factors that contribute to pharmaceutical incentivisation, and the methods they deem important to reduce it. The study also includes collaborations with important regulatory bodies such as the PMC and SHCC. These collaborations not only enable the smooth execution of this sensitive study but can further strengthen the impact of the intervention. The study involves a novel approach, including an emotive element to seminars-based intervention. In particular, the video on COI in medical practice as well as religious messages aims at a more impactful message for GPs towards recognition of the negative effect of pharmaceutical incentivisation on patients’ well-being and health. Other creative methods include slogan competition and reinforcement of the message through shortened COI video reminders that aim to keep GPs engaged with messages on medical ethics delivered as part of the intervention. Finally, to the best of our knowledge, the use of SPSR methodology to evaluate GPs’ practices regarding pharmaceutical incentivisation is novel. This assessment method has the potential to minimise bias in collecting information on how GPs react to incentivisation offers following exposure to the intervention on medical ethics.

The study should also be considered in terms of limitations. A smaller number of GPs in the seminars may ignore invitations due to conflicting professional and personal engagements. While this sample distribution is important to measure the impact of the seminars on GPs with similar characteristics (ie, ethnic identities, sociocultural background and financial status), it can potentially enable information contamination across the groups. The fact that in Pakistan the proportion of female physicians in private primary care clinics is low. As such the insights into the difference in knowledge, attitudes and practices regarding medical ethics between male and female participants provided by our analysis may be limited. Finally, to limit the risk of SPSRs being identified as simulators, each SPSR will be limited to only two visits to each GP clinic.

We hope the study results will help us understand whether emotive seminars may be a useful way to address the issue of pharmaceutical incentivisation in Pakistan. The postseminars qualitative interviews with GP can in particular be of great importance in determining which aspects of ethics-related CME seminars need improvement or greater emphasis. Regulatory bodies including PMC and SHCC can potentially use these findings to introduce new programmes for educating GPs on professional ethics in medical practice. Finally, the study can also potentially shape future interventions to help improve GPs’ practices.

## Supplementary Material

Reviewer comments

Author's manuscript
