# Supplementary material for: Impact of a multifaceted intervention on physicians’ knowledge, attitudes and practices in relation to pharmaceutical incentivisation: protocol for a randomised control trial
Source: BMJ Open. 2022 Nov 4;12(11):e067233. doi: 10.1136/bmjopen-2022-067233 (PMC9639112; doi:10.1136/bmjopen-2022-067233)
Supplement: Supplementary data [file bmjopen-2022-067233supp001.pdf]

Supplementary File 1

PRESCRIBING PRACTICES IN PLURALISTIC HEALTH SYSTEMS IN PAKISTAN
Aga Khan University, Pakistan
Study ERC # 2021-6781-19734

Principal investigators: Dr. Sadia Shakoor, the Department of Pathology and Laboratory Medicine, and Dr Mishal Khan, London School of Hygiene and Tropical Medicine.

Co-Principal Investigators: Dr Rumina Hasan, Dr Naveed Noor, the Department of Pathology and Laboratory Medicine, Dr Sameen Siddiqui, and Dr Rehana Siddiqui, the Department of Community Health Sciences, Aga Khan University.

Institute: The Aga Khan University

INFORMATION SHEET

We would like to invite you to take part in a research study. Joining the study is entirely up to you. Before you decide, you need to understand why the research is being done and what it would involve. One of our team will go through this information sheet with you and answer any questions you may have.

Who is conducting this study?
We are a team of researchers from the London School of Hygiene and Tropical Medicine (LSHTM), and the Aga Khan University (AKU). The study is funded by the UK research councils and the sponsor is LSHTM.

This study has been reviewed and given favourable opinion by the LSHTM (UK) and AKU (Pakistan) ethics Committee (#1234 & # 2021-6781-19734).

Why are we conducting this research?
We are conducting a study that seeks to assess the impact of an education and motivation-based intervention on the knowledge, attitudes, and practice of doctors in Karachi, Pakistan. Findings will be valuable in Pakistan but also in other countries in Asia and around the world.

What will be done if I take part in this research?
If you choose to participate in this research, we will invite you to a seminar discussing important health-related issues. We are conducting an experimental study through seminars given to two groups of doctors. Each group will be taught different topics/contents related to health. After the seminars, we will explore which group learned more. The purpose of these seminars is to provide doctors with information that can improve their professional practice. You will be asked to complete a short survey before and after the seminar. You might also be selected to participate in a brief interview to give us your feedback on the seminar. Following the seminar, we may send you some reminders of the seminar content through your phone or at your clinic. Finally, within 6 months of the seminar, we will conduct an unannounced assessment through physical visits that will allow us to provide you feedback to enhance your professional practice. The assessment will also allow us to examine if our seminar was effective and to find ways to improve its delivery in the future.

Noor MN, *et al. BMJ Open* 2022; 12:e067233. doi: 10.1136/bmjopen-2022-067233

Supplementary File 1

Why have I been selected?
In this study, the participants we are approaching are qualified doctors working as general practitioners (GPs) in Karachi. We are collaborating with the Sindh Healthcare Commission and have randomly selected approximately 300 doctors using a list of private clinics that they maintain.

How shall relevant audiences get to know the results of the study?
The research findings will be published in academic journals and presented at academic conferences and seminars (in Pakistan and internationally) so that doctors and other public health professionals can learn about the study findings. A report and presentation will be made to policy makers in Pakistan.

Are there any disadvantages or advantages involved in taking part?

There only individual benefits to participating is the opportunity to improve your knowledge and professional practice. Your participation may have wider benefits as it will help us to design materials or policies that might improve medical practice. Participating in this research should not cause any discomfort or hazards and you can stop participating at any time by letting a member of the research team know. Any feedback provided to you will be respectful and confidential.

Who will have access to the information I give?
We will not share individual information about you or other participants with anyone beyond our research team. Instead, the knowledge gained from this research will be shared in summary form (without revealing your name or personal details) with organizations or individuals that will find the information useful in improving policy.

We will ask for your consent specifically on whether

a) you are willing to participate in the research
b) whether you agree for us to enter the information you provide into a database that will allow it to be used for future research (without contacting you again)

How will my privacy and the confidentiality of my research records be protected?
Personal information will be used for introductory and familiarity purposes alone. Personal information will never be used in a publication or presentation. All information collected about you will be kept private, safe and secure. Only the study staff who check that the study is being carried out properly will be allowed to look at information about you. All your research data will be coded, meaning that each participant will only be identified with a number. Any data placed in a database or shared with other members of the research team will only contain information that cannot be traced back to you.
Your personal details, meaning your name and other identifiable information, will be kept in a different safe place to the other study information and will be destroyed within 10 years of the end of the study. At the end of the project, the study data will be archived on LSHTM's Secure Server. The data will be made available to other researchers worldwide for research and to improve medical knowledge and patient care. Your personal information will not be included and there is no way that you can be identified.

Where can I find out more about how my information is used?
- At https://www.lshtm.ac.uk/files/research-participant-privacy-notice.pdf
- by asking one of the research team
- by sending an email to DPO@lshtm.ac.uk

Supplementary File 1

Can I change my mind or refuse to participate?
Participation in this study is voluntary. You are free to decide if you want to take part or not. If you do agree, you can change your mind at any time. You can refuse to answer any specific questions or stop participating in the intervention at any time. If you choose not to answer a question, or even decide not to participate in the study at all, it will not affect you in any way today or in the future. If you stop being part of the study at any time, we will keep information about you that we already have.

What if I have any questions?
If you wish to receive further information on the research project, please do not hesitate to contact members of the research team, namely the following:

PI: Dr Sadia Shakoor, sadia.shakoor@aku.edu
CO-PI: Dr Rumina Hasan, rumina.hasan@aku.edu
CO-PI: Dr Naveed Noor, naveed.noor@aku.edu
+92 312 7275661

Noor MN, *et al. BMJ Open* 2022; 12:e067233. doi: 10.1136/bmjopen-2022-067233

Supplementary File 1

CONSENT FORM

I hereby acknowledge that:

1. My signature, as witnessed by the Consent Taker, is my acknowledgement that I have agreed to participate in the interview.
2. I have received a copy of an information sheet that explains my role in this research. I understand its contents and agree to participate in this research.
3. I can withdraw from the research at any point of time
4. I consent / do not consent* to have the coded data made available for future research by putting it into a data repository
5. I agree / do not agree* to audio-recording of my participation in the research.
6. I agree/do not agree* for verbatim quotes to be used without identifying me.

*please circle as appropriate

This research has been explained to me in _________________ (language) on _______ (date).

____
Name and Signature (Participant) Date

____
Name and Signature (Consent Taker) Date
