# Supplementary material for: Impact of a multifaceted intervention on physicians’ knowledge, attitudes and practices in relation to pharmaceutical incentivisation: protocol for a randomised control trial
Source: BMJ Open. 2022 Nov 4;12(11):e067233. doi: 10.1136/bmjopen-2022-067233 (PMC9639112; doi:10.1136/bmjopen-2022-067233)
Supplement: Author's manuscript [file bmjopen-2022-067233.draft_revisions.pdf]

BMJ Open is committed to open peer review. As part of this commitment we make the peer review history of every article we publish publicly available.

When an article is published we post the peer reviewers' comments and the authors' responses online. We also post the versions of the paper that were used during peer review. These are the versions that the peer review comments apply to.

The versions of the paper that follow are the versions that were submitted during the peer review process. They are not the versions of record or the final published versions. They should not be cited or distributed as the published version of this manuscript.

BMJ Open is an open access journal and the full, final, typeset and author-corrected version of record of the manuscript is available on our site with no access controls, subscription charges or pay-per-view fees (http://bmjopen.bmj.com).

If you have any questions on BMJ Open's open peer review process please email info.bmjopen@bmj.com

BMJ Open

The impact of a multifaceted intervention on physicians' knowledge, attitudes, and practices in relation to pharmaceutical incentivisation: protocol for a randomised control trial

Journal:	*BMJ Open*
Manuscript ID	bmjopen-2022-067233
Article Type:	Protocol
Date Submitted by the Author:	05-Aug-2022

Complete List of Authors:	Noor, Muhammad Naveed; The Aga Khan University, Department of pathology and laboratory medicine<br>Khan, Mishal; London School of Hygiene and Tropical Medicine,<br>Rahman-Shepherd, Afifah; London School of Hygiene and Tropical Medicine Faculty of Public Health and Policy<br>Siddiqui, Amna Rehana; Aga Khan University, Community Health Sciences<br>Khan, Sabeen; The Aga Khan University<br>Azam, Iqbal; The Aga Khan University, Community Health Sciences<br>Shakoor, Sadia; Aga Khan University<br>Hasan, Rumina; The Aga Khan University Hospital Main Campus Karachi; London School of Hygiene and Tropical Medicine Faculty of Public Health and Policy

Keywords:	Protocols & guidelines < HEALTH SERVICES ADMINISTRATION & MANAGEMENT, Health policy < HEALTH SERVICES ADMINISTRATION & MANAGEMENT, MEDICAL ETHICS, EDUCATION & TRAINING (see Medical Education & Training), Quality in health care < HEALTH SERVICES ADMINISTRATION & MANAGEMENT, Public health < INFECTIOUS DISEASES

SCHOLARONE™
Manuscripts

The impact of a multifaceted intervention on physicians' knowledge, attitudes, and practices in relation to pharmaceutical incentivisation: protocol for a randomised control trial

Muhammad Naveed Noor[a]*, Mishal Khan[c]*, Afifah Rahman-Shepherd#, Rehana Siddiqui[b]#, Sabeen Sharif[a], Iqbal Azam[b], Sadia Shakoor[a], Rumina Hasan[a,b,d]+

*Joint first authors

#Joint second authors

+ Corresponding author

Contact: Dr Rumina Hasan, rumina.hasan@aku.edu

a:Department of Pathology & Laboratory Medicine, Aga Khan University, Karachi, Pakistan; b:Department of Community Health Sciences, Aga Khan University, Karachi, Pakistan; c:Department of Global Health & Development, London School of Hygiene & Tropical Medicine, London, UK, d: Faculty of Infectious and Tropical Diseases, London School of Hygiene and Tropical Medicine, London, UK.

For peer review only - http://bmjopen.bmj.com/site/about/guidelines.xhtml

For peer review only

The impact of a multifaceted intervention on physicians' knowledge, attitudes, and practices in relation to pharmaceutical incentivisation: protocol for a randomised control trial

Abstract

Introduction: In settings where the private sector constitutes a larger part of the health system, profit-gathering can take primacy over patients' wellbeing. In their interactions with pharmaceutical companies, private general practitioners (GPs) can experience a conflict of interest (COI), a situation whereby the impartiality of GPs' professional decision-making may be influenced by secondary interests such as financial gains from prescribing specific pharmaceutical brands.

Methods and analysis: This study is a randomised control trial (RCT) to assess the impact of a multifaceted intervention on GPs' medical practice. The study sample consists of 419 registered GPs who own/work in private clinics and will be randomly assigned to intervention and control groups. The intervention group GPs will be exposed to emotive and educational seminars on medical ethics, whereas control group GPs will be given seminars on general medical topics. The primary outcome measure will be GPs' prescribing practices, whereas the secondary outcome measures will be their knowledge and attitudes regarding COI that arises from pharmaceutical incentivisation. In addition to a novel standardised pharmaceutical representatives (SPSR) method, in which field researchers will simulate pharmaceutical marketing with GPs, pre-and post-surveys, and qualitative interviewing will be performed to collect data on GPs' knowledge, attitudes and practices in relation to COI linked with pharmaceutical incentives. Univariate and multivariate statistical analyses will be performed to measure a change in GPs' knowledge, attitudes, and practices, while qualitative analysis will add to our understanding of the quantitative SPSR data.

Ethics and dissemination: Ethics approval has been obtained from the Pakistan National Bioethics Committee (# 4-87/NBC-582/21/1364), the Aga Khan University (# 2020-4759-1129) and the London School of Hygiene and Tropical Medicine (# 26506). We will release results within 6 to 9 months of the study's completion.

Trial registration number: ISRCTN12294839

Keywords: protocols and guidelines, health policy, medical ethics, education and training, quality in healthcare, public health

Strengths and limitations of the study

1. This is the first study to employ a randomised control trial (RCT) method to assess the impact of a multifaceted intervention on GPs' knowledge, attitudes, and practices regarding COI in medical practice arising from pharmaceutical incentivisation.
2. The study involves the co-development of the intervention through qualitative formative research with GPs, policy actors, and pharmaceutical sales representatives (PSRs).

For peer review only - http://bmjopen.bmj.com/site/about/guidelines.xhtml

3. The study uses a novel standardised pharmaceutical sales representative (SPSR) method to assess the impact of the intervention.
4. The study will inform further research and interventions to reduce the conflict of interest (COI) in medical practice.
5. The GPs included in the study have been randomly selected. However, due to certain sociocultural factors, female doctors are less likely to work as primary care providers in Pakistan. Therefore, the study sample encompasses a greater proportion of male GPs.

Introduction

The pharmaceutical industry is often a vital source of information for general practitioners (GPs) to learn about pharmaceutical products. This information helps GPs make informed choices and decisions in relation to patients' diverse health needs [1]. Nevertheless, the relationship between the pharmaceutical industry and GPs can negatively affect patients' health and wellbeing, if/when incentives are mobilised to maximise profits through inappropriate prescribing [2]. As a result of pharmaceutical incentivisation, GPs may experience a conflict of interest (COI) - a situation where GPs' professional judgment is influenced by a secondary interest, such as financial gain, leading to a decision that conflicts with their interpretation of the patient's best interest [3, 4]. There is evidence that GPs who engage in pharmaceutical incentivisation are likely to prescribe unnecessary and/or more expensive medicines to patients, further contributing to an increased financial burden on them [5, 6]. In addition, pharmaceutical incentivisation has important implications for antimicrobial resistance (AMR), as to achieve pharmaceutical targets, GPs may prescribe antibiotics to patients with common self-resolving ailments like cold and flu [7].

In Pakistan, the health system is comprised of public and private sectors where a large proportion of patients access healthcare from private GPs [8]. At the same time, the country is home to over 600 pharmaceutical companies including multinational, national, and franchise-based local companies [9]. To compete in the market, pharmaceutical companies use

For peer review only - http://bmjopen.bmj.com/site/about/guidelines.xhtml

incentivisation as a major tool to maximise profits from GPs' prescriptions [10]. On the other hand, in Pakistan, poverty represents one of the key problems that people and the inextricable link between poverty and a lack of education and health awareness gives perfect grounds to the pharmaceutical industry and GPs to capitalise on patients' weaknesses to maximise profits from pharmaceutical products, further exacerbating problems for patients [11, 12].

Our previous research in Pakistan helped us identify three interlinked health system constraints that shape private GPs' decisions on receiving personal benefits from prescribing antibiotics [13-17]. First, insufficient income generation from patient consultations alone - as patients are unwilling to spend substantial sums on medical advice without a prescription - resulted in a reliance on other sources of income [16]. Second, limited opportunities for continuing medical education other than from pharmaceutical companies may lead to a close relationship with pharmaceutical sales representatives [14]. Third, inadequate access to diagnostic tools to establish the clinical need for antibiotics, making it easier for doctors to justify defensive over-prescribing [14]. We also found that private doctors engage in activities such as generating extra income from receiving monetary or non-monetary incentives, and sponsorships to attend academic events from pharmaceutical companies in return for meeting sales targets for specific medicines [14]. These behaviours of private physicians stem from deeply embedded dynamics of pluralistic health systems. Hence, not only did these studies provide rich insights into critical health system and policy dynamics, but also helped to identify the issue of COI in medical practice and how this contributes to unnecessary prescribing of medicines in primary care settings.

Therefore, to improve the quality of primary care, actions are required that address pharmaceutical incentivisation [18, 19]. However, major impediments to progress on this front

For peer review only - http://bmjopen.bmj.com/site/about/guidelines.xhtml

include limited assessment of interventions for improving the quality of care delivered by private GPs [20, 21], and a marked absence of attention by researchers and policymakers as to the role of political, social, economic and cultural factors that influence the effectiveness and scalability of interventions [22]. Furthermore, prohibiting or constraining private physicians through the enforcement of regulations may fail because of a lack of political and social support and enforcement capacity, as well as insufficient public sector capacity to provide care [20]. Training interventions focusing on increasing technical knowledge and skills are the most common approach used to improve the quality of care [23, 24]. Our previous research also suggests that an intervention that can enhance GPs' understanding of COI in medical practice might be useful to enable them to recognise potential actions that threaten professional ethics in their medical practice while interacting with pharmaceutical sales representatives (PSRs) [14, 16]. It is clear, however, from consistent evidence on the 'know-do' gap that COI related to profit generation from medicine sales plays a critical role in prescribing decisions, as do values associated with professional ethics and altruism [25, 26]. To improve GPs' understanding of COI in medical practice, we aim to develop an intervention based on emotive and educational seminars and to test their impact on private GPs' knowledge, attitudes, and practices in relation to professional ethics in medical practice.

Study objectives

The overarching aim of our study is to assess the impact of a multifaceted intervention for improving GPs' prescribing practices in relation to COI linked with pharmaceutical incentivisation. The specific objectives of the study are to:

- Alter GPs' prescribing practice in response to pharmaceutical incentives.

For peer review only - http://bmjopen.bmj.com/site/about/guidelines.xhtml

- Improve their knowledge of professional ethics in medical practice.
- Shift their attitudes to give primacy to patients' wellbeing rather than pharmaceutical incentives.

Methods

We plan to complete the study in three phases: formative, intervention, and evaluation. The study is being undertaken in Karachi which is the largest city by geographical area and population size in Pakistan [27]. In 2021, we completed the formative phase of the study, whereas the intervention and evaluation phases will be completed in 2022.

Formative work

We used semi-structured interviews to obtain information from GPs, policy actors, and PSRs about factors that lead to COI arising from pharmaceutical incentivisation. Individuals from regulatory institutions, professional medical associations, and health communication/media consultants were deemed, policy actors. One of the key objectives of the formative work was to co-develop a multifaceted intervention that could improve GPs' knowledge, attitude, and practices about professional ethics in medical practice. Preliminary analysis of our formative work suggests that in Pakistan, professional ethics in medical practice have gradually changed and engaging with pharmaceutical incentivisation has become a norm. Competition in the pharmaceutical market, GPs' desire to maximise income, combined with weaknesses in health regulation produce a context where an unethical relationship between the pharmaceutical industry and GPs develop and is sustained. Based on our findings, recommendations from study participants, and existing research, a multifaceted intervention has been developed in which, GPs

For peer review only - http://bmjopen.bmj.com/site/about/guidelines.xhtml

have been invited to seminars to get exposure to messages on medical ethics.

Intervention

Our intervention is based on multifaceted seminars. As indicated in figure 1, the intervention group has been given seminars on ethics in relation to COI linked with pharmaceutical incentivisation, while the control group has received seminars on clinical issues such as thalassemia and anemia. The intervention seminars have been developed in four major phases. First, international and national guidelines have been reviewed to develop a brief document on professional ethics in medical practice. The document covers several issues including pharmaceutical incentivisation in the form of money, other material/nonmaterial benefits, and the misuse of the medical license. The document has been endorsed by regulatory bodies at the federal and provincial levels including the Pakistan Medical Commission (PMC) and the Sindh Healthcare Commission (SHCC). Second, in collaboration with media/communication experts, a filmmaker has been hired to produce an emotive video highlighting the effects of profit-led prescribing on patients' wellbeing. Third, GPs have been invited to attend seminars on professional ethics in medical practice and on managing anaemia and thalassemia in primary care settings. The intervention group GPs has also been sent the PMC- and SHCC-endorsed brief on ethics in medical practice (reinforcement) and the shortened version of the emotive video (reminder).

The control seminars have been developed in three phases. First, we identify experts in thalassemia and anemia research and take them on board to teach these topics to the control group GPs. Second, in collaboration with the experts, we develop a short educational video on thalassemia and handouts on the management of thalassemia and anemia in primary care

For peer review only - http://bmjopen.bmj.com/site/about/guidelines.xhtml

settings. Third, we invite the control group GPs to attend the seminars.

Outcome measures

The primary outcome measure of the study is the number of GPs who refuse to take unethical pharmaceutical incentives from SPSRs. The secondary outcomes include GP's change in knowledge and attitudes about professional ethics in medical practice.

Participants

Private GPs, registered with the PMC and the SHCC are deemed eligible to participate in the study. GPs outside Karachi, those working in welfare clinics, dentists, consultants, and unqualified healthcare providers are excluded from the study.

Sample size

For the present study, a list of 1695 GPs, obtained from the SHCC has been considered a sample universe. From it, 419 GPs have been sampled using a systematic sampling technique. In our literature review, we identify no relevant surveys of private GPs in Pakistan, so we use data from two studies on medical students and trainee doctors, to calculate the sample size for our study. One survey of medical students shows that 81% favoured pharmaceutical sponsorship of events at medical colleges [28] and the other study on trainee doctors in public hospitals indicates that 57% did not know any code of medical ethics [29]. Our qualitative research with private doctors also shows that receiving benefits for prescribing is very common (accepted for publications in Health Sociology Review [# RHSR-2022-0065] and PLOS Global Public Health [# PGPH-D-22-00588]). We, therefore, estimate that 80% of private GPs in our sample will be classified as 'supporting pharmaceutical incentivisation' and assume that our intervention shifts this to 65%,

For peer review only - http://bmjopen.bmj.com/site/about/guidelines.xhtml

in line with changes observed in the few interventional studies addressing COI in medical professionals [30, 31]. Using these assumptions, a sample size of between 130-135 per group gives a power of 80% to detect a difference owing to the intervention. As indicated in figure 2, the sample of 419 GPs was extracted in three steps. First, the entire list is screened to exclude 510 healthcare providers who do not meet our eligibility criteria Second, information validation is performed by calling the remaining 1185 healthcare providers to verify important information such as clinic addresses, registration status, level of education, and whether they are active/inactive in medical practice, further leading to the exclusion of 764 names. As part of a baseline survey on GPs' knowledge and attitudes about ethics in medical practice, 3 GPs have been further excluded from the sample who refuse to participate in the study.

Randomisation

The 419 GPs who completed the baseline survey and consented to attend the seminars have been assigned to control and intervention groups, using a simple randomisation technique. Each GP in the list has been first assigned a unique identification number, following the sorting of the list with respect to six districts in Karachi, namely, East, West, Central, South, Korangi, and Malir. Each GP has then been assigned a random number using the excel spreadsheet. Third, within each district, GPs are assigned to control and intervention groups by sorting the list. The intervention and control groups comprised 210 and 209 GPs, of which, 135 and 132 GPs have attended the seminars. To prevent bias, we have collaborated with a statistician outside the research team to perform the randomisation process. Participants and data collectors are blinded in relation to GPs' allocation to control and intervention groups.

For peer review only - http://bmjopen.bmj.com/site/about/guidelines.xhtml

Data collection

To measure the impact of the intervention, our data collection is underway, and we anticipate completing it in September/October 2022. We adopt a multi-method approach to collect data on GPs' knowledge, attitudes, and practices in relation to COI linked with pharmaceutical incentivisation. Pre- and post-intervention surveys will be used to assess a change in GPs' knowledge of professional medical ethics and attitudes about engaging in pharmaceutical incentivisation. To collect information about their practices with respect to taking incentives for prescribing, a novel SPSR method will be used, in which trained field researchers will visit the GP clinics as pharmaceutical marketers to assess GPs' reactions to pharmaceutical incentivisation offers. The SPSRs will recite a script (prepared by the research team) to introduce themselves and the imaginary pharmaceutical company and will market antibiotics commonly prescribed. In this process, the SPSRs make a clear incentivisation offer to GPs for prescribing the medicines and assess whether GPs accept/reject the offer. Since the SPSRs will be representing new pharmaceutical companies and there will be less familiarity between them and GPs, it is anticipated that there can be GPs' responses, difficult to classify in terms of acceptance or rejection. Therefore, situations in which GPs ignore the SPSR offers or ask them to come back later will be classified as "undetermined". The SPSRs will complete an electronic questionnaire after each GP visit to record information such as GPs' demographic and clinical characteristics, and their responses to the incentivisation offer. To ensure the data quality, the research team will put several measures in place: recruiting field supervisors who will accompany the SPSRs to GP clinics and ensure the SPSRs' responses in the questionnaire reflect the interactions with GPs, WhatsApp voice notes to the research team to summarise the interactions and monitoring the SPSR data considering the voice notes as a reference. The questionnaire will be analysed by

For peer review only - http://bmjopen.bmj.com/site/about/guidelines.xhtml

research team members (also blinded to the allocation of GPs), who will assign a binary outcome for each GP such as “refused incentives immediately”, “refused incentives after being offered”, “asked for and accepted an incentive”, “offered, and accepted an incentive”, “undetermined/undecided”. To prevent the SPSR assessment from being discovered as a simulation that not only can risk the intervention assessment but the SPSRs, in terms of their physical safety, each GP is paid a maximum of two visits. It is also important to note that the SPSRs are blinded to the allocation of GPs.

GPs have been made aware, through the consent procedure in the baseline survey, that their behaviour will be assessed covertly at some stage over 6 months but will not know details of the assessment to avoid the Hawthorne effect. We considered this novel SPSR approach to be superior to a revealed preferences approach (e.g., based on medical records) because records of prescribing by private physicians usually do not exist or are of poor quality in Pakistan, and to a stated preference design as it would rely on honest reporting of behaviour that is highlighted as unethical in the COI presentation.

Finally, to explain the undecided/undetermined SPSR assessment outcomes, we will conduct 20-25 qualitative interviews with GPs and get information on potentially unclear SPSR assessment outcomes.

Data analysis

The primary outcome will be assessed by comparing the proportion of GPs classified as ‘yes’ to accepting an unethical benefit (according to the regulator’s guidelines) for prescribing the SPSR’s antibiotic between the control and intervention groups. The proportions will be compared using a two-proportion z-test. Secondary outcomes such as knowledge and attitude

For peer review only - http://bmjopen.bmj.com/site/about/guidelines.xhtml

shifts will be assessed by comparing mean scores in survey responses of control and intervention groups using unpaired t-test and proportions scores in survey responses using the z-test.

Ethics and dissemination

Ethics approval has been obtained from the Pakistan National Bioethics Committee (# 4-87/NBC-582/21/1364), the Aga Khan University (# 2020-4759-1129) and the London School of Hygiene and Tropical Medicine (# 26506). Key ethical guidelines about informed consent, voluntary participation, and maintaining participants' confidentiality will be observed throughout the research process.

Study results will be disseminated through open-access peer-reviewed publications targeting health promotion professionals, policymakers, and researchers. The study will also be presented at academic conferences in medicine and public health.

Discussion

Our study is the first to evaluate the impact of a multifaceted intervention on GPs' knowledge, attitudes, and practices in relation to COI linked with pharmaceutical incentives: an area that has not been well studied in Pakistan. It may guide the design of further studies and interventions to improve prescribing practices in private primary care settings. We have attempted to develop a comprehensive approach to analyse the issue of pharmaceutical incentivisation and to suggest potential ways to address it. Our main objective is to examine the relationship between the pharmaceutical industry and GPs and to develop and test an emotive seminar-based intervention to improve GPs' knowledge, attitudes, and practices about professional ethics in medical practice.

For peer review only - http://bmjopen.bmj.com/site/about/guidelines.xhtml

The strengths of the study include the fact that it systematically uses different levels of investigation including formative work to understand how GPs, policy actors, and PSRs conceptualise COI in medical practice, factors that contribute to pharmaceutical incentivisation, and the methods they deem important to reduce it. The study also includes collaborations with important regulatory bodies such as the PMC and SHCC. These collaborations not only enable the smooth execution of this sensitive study but can further strengthen the impact of the intervention. The study involves a novel approach, including an emotive element to seminars-based intervention. In particular, the video on COI in medical practice as well as religious messages aims at a more impactful message for GPs towards recognition of the negative effect of pharmaceutical incentivisation on patients' wellbeing and health. Other creative methods include slogan competition and reinforcement of the message through shortened COI video reminders that aim to keep GPs engaged with messages on medical ethics delivered as part of the intervention. Finally, to the best of our knowledge, the use of SPSR methodology to evaluate GPs' practices regarding pharmaceutical incentivisation is novel. This assessment method has the potential to minimise bias in collecting information on how GPs react to incentivisation offers following exposure to the intervention on medical ethics.

The study should also be considered in terms of limitations. A smaller number of GPs in the seminars may ignore invitations due to conflicting professional and personal engagements. While this sample distribution is important to measure the impact of the seminars on GPs with similar characteristics (i.e., ethnic identities, sociocultural background, and financial status), it can potentially enable information contamination across the groups. The fact that in Pakistan the proportion of female physicians in private primary care clinics is low. As such the insights into the difference in knowledge, attitudes, and practices regarding medical ethics between male and

For peer review only - http://bmjopen.bmj.com/site/about/guidelines.xhtml

female participants provided by our analysis may be limited. Finally, to limit the risk of SPSRs being identified as simulators, each SPSR was limited to only 2 visits to each GP clinic.

We hope the study results will help us understand whether emotive seminars may be a useful way to address the issue of pharmaceutical incentivisation in Pakistan. The post-seminars qualitative interviews with GP can in particular be of great importance in determining which aspects of ethics-related continuing medical education seminars need improvement or greater emphasis. Regulatory bodies including PMC and SHCC can potentially use these findings to introduce new programs for educating GPs on professional ethics in medical practice. Finally, the study can also potentially shape future interventions to help improve GPs' practices.

Acknowledgements

We would like to acknowledge support from various people and institutions including Dr Robyna Irshad Khan and Dr Natasha Ali (Associate Deans at AKU), Dr Sameen Siddiqi (Chair, Community Health Sciences, AKU), the PMC, the SHCC, and the Centre for Biomedical Culture and Ethics (CBEC). We are also thankful to the staff at the AKU's microscopy laboratory to help us with managing the logistics of the seminars.

Authors' contributions

MK and SS conceptualised the study. SS, RH, MNN, and AmnaRS contributed to the study design. MNN contributed to the drafting and writing of this protocol. MK, RH, SS, AfifahRS and AmnaRS contributed equally through critically reviewing and revising the proposal. AfifahRS developed the flowchart indicating the sampling and randomisation process. IA performed randomisation. SSK assisted in intervention design and delivery.

For peer review only

Funding statement

This work is supported by the UK Research and Innovation (UKRI) under grant number MR/T02349X/1.

Competing interests statement

The authors have no competing interests to declare.

For peer review only

References

1. Yager, J. and R.E. Feinstein, *Medical education meets pharma: moving ahead.* Academic Psychiatry, 2010. **34**(2): p. 92-97.
2. Sismondo, S., *Epistemic corruption, the pharmaceutical industry, and the body of medical science.* Frontiers in Research Metrics and Analytics, 2021. **6**: p. 614013.
3. Yousafzai, A., *Corruption in medical practice: Where do we stand?* 2015. **J Ayub Med Coll Abbottabad 2015;27(3)**.
4. Trap, B., *Practices of dispensing doctors-drug use and health economics*, in *International Conferences on Improving Use of Medicines*. 1997, Zimbabwe Essential Drugs Action Programme, Ministry of Health & Child Welfare.
5. Khazzaka, M., *Pharmaceutical marketing strategies' influence on physicians' prescribing pattern in Lebanon: ethics, gifts, and samples.* BMC health services research, 2019. **19**(1): p. 1-11.
6. Naqvi, A., et al., *Report: Interactions and conflicts of interests between prescribers and medical sales representatives (MSRs) regarding prescribing and drug promotion practices in Karachi, Pakistan.* Pakistan journal of pharmaceutical sciences, 2019. **32**(2): p. 687-695.
7. Morel, C.M., et al., *Industry incentives and antibiotic resistance: an introduction to the antibiotic susceptibility bonus.* The Journal of Antibiotics, 2020. **73**(7): p. 421-428.
8. Kurji, Z., Z.S. Premani, and Y. Mithani, *Analysis of the health care system of Pakistan: lessons learnt and way forward.* J Ayub Med Coll Abbottabad, 2016. **28**(3): p. 601.
9. Mehmood, K.K., F. Sonia, and A. Umar, *Impact of Organic Structure on Competitive Performance of Pharmaceutical Companies in Pakistan: Study of Mediating Roles.* Pakistan Journal of Social Sciences (PJSS), 2016. **36**(2).
10. Gul, R., et al., *Perceptions of and barriers to ethical promotion of pharmaceuticals in Pakistan: perspectives of medical representatives and doctors.* BMC medical ethics, 2021. **22**(1): p. 1-16.
11. Saleem, H., M.S. Shabbir, and B. Khan, *Re-examining multidimensional poverty in Pakistan: A new assessment of regional variations.* Global Business Review, 2021. **22**(6): p. 1441-1458.
12. Arsani, A.M., B. Ario, and A.F. Ramadhan, *Impact of education on poverty and health: Evidence from Indonesia.* Economics Development Analysis Journal, 2020. **9**(1): p. 87-96.
13. Aftab, W., et al., *Variations in regulations to control standards for training and licensing of physicians: a multi-country comparison.* Human resources for health, 2021. **19**(1): p. 1-10.
14. Khan, M.S., et al., *What are the barriers to implementing national antimicrobial resistance action plans? A novel mixed-methods policy analysis in Pakistan.* Health policy and planning, 2020. **35**(8): p. 973-982.

15. Khan, M.S., et al., *'LMICs as reservoirs of AMR': a comparative analysis of policy discourse on antimicrobial resistance with reference to Pakistan.* Health Policy and Planning, 2019. **34**(3): p. 178-187.
16. Khan, M., et al., *Should performance-based incentives be used to motivate health care providers? Views of health sector managers in Cambodia, China and Pakistan.* Health Economics, Policy and Law, 2020. **15**(2): p. 247-260.
17. Khan, M.S., et al., *How do external donors influence national health policy processes? Experiences of domestic policy actors in Cambodia and Pakistan.* Health policy and planning, 2018. **33**(2): p. 215-223.
18. Das, J. and J. Hammer, *Quality of Primary Care in Low-Income Countries: Facts and Economics.* Annual Review of Economics, 2014. **6**(1): p. 525-553.
19. Morgan, R., T. Ensor, and H. Waters, *Performance of private sector health care: implications for universal health coverage.* The Lancet, 2016. **388**(10044): p. 606-612.
20. Montagu, D. and C. Goodman, *Prohibit, constrain, encourage, or purchase: how should we engage with the private health-care sector?* Lancet, 2016. **388**(10044): p. 613-21.
21. Shah, N.M., W.R. Brieger, and D.H. Peters, *Can interventions improve health services from informal private providers in low and middle-income countries?: a comprehensive review of the literature.* Health Policy Plan, 2011. **26**(4): p. 275-87.
22. Ghaffar, A., et al., *Where is the policy in health policy and systems research agenda?* Bull World Health Organ, 2016. **94**(4): p. 306-8.
23. Chakraborty, S., S.A. D'Souza, and R.S. Northrup, *Improving private practitioner care of sick children: testing new approaches in rural Bihar.* Health Policy Plan, 2000. **15**(4): p. 400-7.
24. Marsh, V.M., et al., *Improving malaria home treatment by training drug retailers in rural Kenya.* Trop Med Int Health, 2004. **9**(4): p. 451-60.
25. Chuc, N.T., et al., *Management of childhood acute respiratory infections at private pharmacies in Vietnam.* Ann Pharmacother, 2001. **35**(10): p. 1283-8.
26. Leonard, K.L. and M.C. Masatu, *Professionalism and the know-do gap: exploring intrinsic motivation among health workers in Tanzania.* Health Economics, 2010. **19**(12): p. 1461-1477.
27. Yusuf, H., *Conflict dynamics in Karachi*. Vol. 19. 2012: US Institute of Peace Washington.
28. Siddiqui, U.T., et al., *Attitudes of medical students towards incentives offered by pharmaceutical companies-perspective from a developing nation-a cross sectional study.* BMC medical ethics, 2014. **15**(1): p. 1-10.
29. Imran, N., et al., *HEALTH ETHICS EDUCATION: KNOWLEDGE, ATTITUDES AND PRACTICE OF HEALTHCARE ETHICS AMONG INTERNS AND RESIDENTS IN PAKISTAN.* JPMI: Journal of Postgraduate Medical Institute, 2014. **28**(4).
30. Thirunavukarasu, P., et al., *Educational intervention is effective in improving knowledge and confidence in surgical ethics—a prospective study.* The American Journal of Surgery, 2010. **200**(5): p. 665-669.
31. Zakaria, A.M., W.F. Sleem, and A.M. Seada, *Effectiveness of ethical issues teaching program on knowledge, ethical behavior and ethical stress among nurses.* Journal of Nursing Education and Practice, 2016. **6**(7): p. 125.

For peer review only - http://bmjopen.bmj.com/site/about/guidelines.xhtml

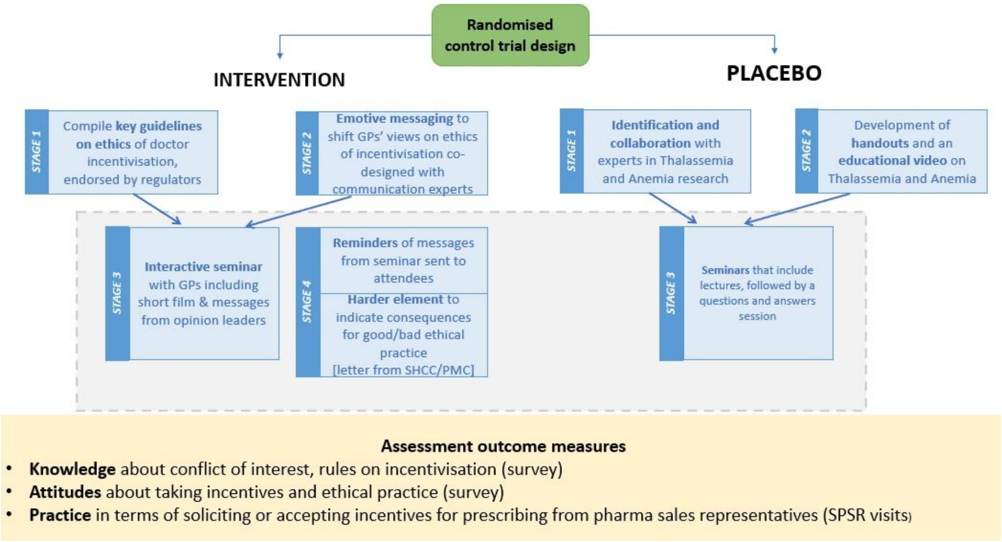

Diagram for intervention and control group seminars

221x120mm (120 x 120 DPI)

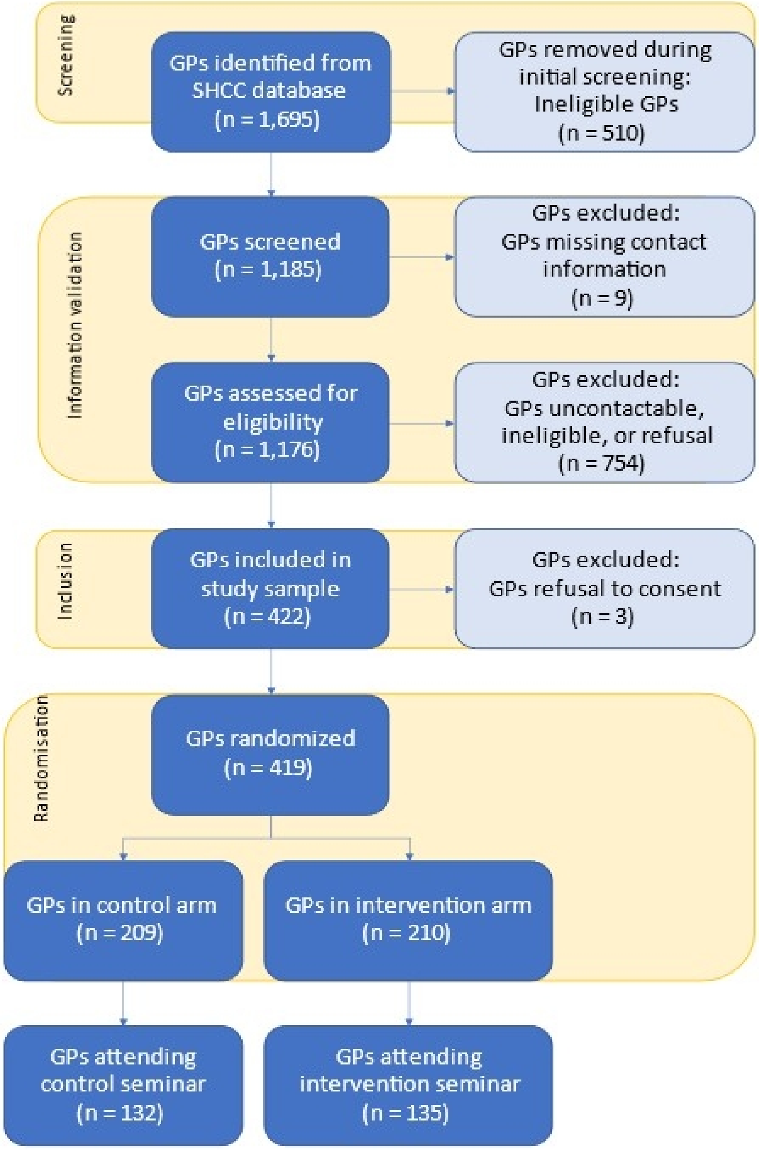

Flowchart of the sampling and randomisation process

125x186mm (96 x 96 DPI)

CONSORT 2010 checklist of information to include when reporting a randomised trial*

Section/Topic	Item No	Checklist item	Reported on page No
**Title and abstract**			
	1a	Identification as a randomised trial in the title	2
	1b	Structured summary of trial design, methods, results, and conclusions (for specific guidance see CONSORT for abstracts)	2
**Introduction**			
Background and objectives	2a	Scientific background and explanation of rationale	3-5
	2b	Specific objectives or hypotheses	5-6
**Methods**			
Trial design	3a	Description of trial design (such as parallel, factorial) including allocation ratio	NA
	3b	Important changes to methods after trial commencement (such as eligibility criteria), with reasons	NA
Participants	4a	Eligibility criteria for participants	6, 9-11
	4b	Settings and locations where the data were collected	
Interventions	5	The interventions for each group with sufficient details to allow replication, including how and when they were actually administered	7-8
Outcomes	6a	Completely defined pre-specified primary and secondary outcome measures, including how and when they were assessed	8
	6b	Any changes to trial outcomes after the trial commenced, with reasons	NA
Sample size	7a	How sample size was determined	8-9
	7b	When applicable, explanation of any interim analyses and stopping guidelines	NA
Randomisation:			
Sequence generation	8a	Method used to generate the random allocation sequence	9
	8b	Type of randomisation; details of any restriction (such as blocking and block size)	9
Allocation concealment mechanism	9	Mechanism used to implement the random allocation sequence (such as sequentially numbered containers), describing any steps taken to conceal the sequence until interventions were assigned	9
Implementation	10	Who generated the random allocation sequence, who enrolled participants, and who assigned participants to interventions	9
Blinding	11a	If done, who was blinded after assignment to interventions (for example, participants, care providers, those	9

For peer review only - http://bmjopen.bmj.com/site/about/guidelines.xhtml
For peer review only

		assessing outcomes) and how	
	11b	If relevant, description of the similarity of interventions	NA
Statistical methods	12a	Statistical methods used to compare groups for primary and secondary outcomes	NA
	12b	Methods for additional analyses, such as subgroup analyses and adjusted analyses	NA
**Results**			
Participant flow (a diagram is strongly recommended)	13a	For each group, the numbers of participants who were randomly assigned, received intended treatment, and were analysed for the primary outcome	NA
	13b	For each group, losses and exclusions after randomisation, together with reasons	NA
Recruitment	14a	Dates defining the periods of recruitment and follow-up	NA
	14b	Why the trial ended or was stopped	NA
Baseline data	15	A table showing baseline demographic and clinical characteristics for each group	NA
Numbers analysed	16	For each group, number of participants (denominator) included in each analysis and whether the analysis was by original assigned groups	NA
Outcomes and estimation	17a	For each primary and secondary outcome, results for each group, and the estimated effect size and its precision (such as 95% confidence interval)	NA
	17b	For binary outcomes, presentation of both absolute and relative effect sizes is recommended	NA
Ancillary analyses	18	Results of any other analyses performed, including subgroup analyses and adjusted analyses, distinguishing pre-specified from exploratory	NA
Harms	19	All important harms or unintended effects in each group (for specific guidance see CONSORT for harms)	NA
**Discussion**			
Limitations	20	Trial limitations, addressing sources of potential bias, imprecision, and, if relevant, multiplicity of analyses	13-14
Generalisability	21	Generalisability (external validity, applicability) of the trial findings	NA
Interpretation	22	Interpretation consistent with results, balancing benefits and harms, and considering other relevant evidence	NA
**Other information**			
Registration	23	Registration number and name of trial registry	2
Protocol	24	Where the full trial protocol can be accessed, if available	NA
Funding	25	Sources of funding and other support (such as supply of drugs), role of funders	15

*We strongly recommend reading this statement in conjunction with the CONSORT 2010 Explanation and Elaboration for important clarifications on all the items. If relevant, we also recommend reading CONSORT extensions for cluster randomised trials, non-inferiority and equivalence trials, non-pharmacological treatments, herbal interventions, and pragmatic trials. Additional extensions are forthcoming: for those and for up to date references relevant to this checklist, see www.consort-statement.org.

For peer review only - http://bmjopen.bmj.com/site/about/guidelines.xhtml

BMJ Open

The impact of a multifaceted intervention on physicians' knowledge, attitudes, and practices in relation to pharmaceutical incentivisation: protocol for a randomised control trial

Journal:	*BMJ Open*
Manuscript ID	bmjopen-2022-067233.R1
Article Type:	Protocol
Date Submitted by the Author:	30-Sep-2022

Complete List of Authors:	Noor, Muhammad Naveed; The Aga Khan University, Department of pathology and laboratory medicine<br>Khan, Mishal; London School of Hygiene and Tropical Medicine,<br>Rahman-Shepherd, Afifah; London School of Hygiene and Tropical Medicine Faculty of Public Health and Policy<br>Siddiqui, Amna Rehana; Aga Khan University, Community Health Sciences<br>Khan, Sabeen; The Aga Khan University<br>Azam, Iqbal; The Aga Khan University, Community Health Sciences<br>Shakoor, Sadia; Aga Khan University<br>Hasan, Rumina; The Aga Khan University Hospital Main Campus Karachi; London School of Hygiene and Tropical Medicine Faculty of Public Health and Policy

**Primary Subject Heading**:	Health services research
Secondary Subject Heading:	Ethics, General practice / Family practice, Health policy, Public health
Keywords:	Protocols & guidelines < HEALTH SERVICES ADMINISTRATION & MANAGEMENT, Health policy < HEALTH SERVICES ADMINISTRATION & MANAGEMENT, MEDICAL ETHICS, EDUCATION & TRAINING (see Medical Education & Training), Quality in health care < HEALTH SERVICES ADMINISTRATION & MANAGEMENT, Public health < INFECTIOUS DISEASES

SCHOLARONE™
Manuscripts

The impact of a multifaceted intervention on physicians' knowledge, attitudes, and practices in relation to pharmaceutical incentivisation: protocol for a randomised control trial

Muhammad Naveed Noor[a]*, Mishal Khan[c]*, Afifah Rahman-Shepherd#, Amna Rehana Siddiqui[b]#, Sabeen Sharif[a], Iqbal Azam[b], Sadia Shakoor[a], Rumina Hasan[a,d]+

*Joint first authors

#Joint second authors

+ Corresponding author

Contact: Dr Rumina Hasan, rumina.hasan@aku.edu

aDepartment of Pathology & Laboratory Medicine, Aga Khan University, Karachi, Pakistan; bDepartment of Community Health Sciences, Aga Khan University, Karachi, Pakistan; cDepartment of Global Health & Development, London School of Hygiene & Tropical Medicine, London, United Kingdom; dFaculty of Infectious and Tropical Disease, London School of Hygiene and Tropical Medicine, London, United Kingdom.

For peer review only - http://bmjopen.bmj.com/site/about/guidelines.xhtml

The impact of a multifaceted intervention on physicians' knowledge, attitudes, and practices in relation to pharmaceutical incentivisation: protocol for a randomised control trial

Abstract

Introduction: In settings where the private sector constitutes a larger part of the health system, profit-gathering can take primacy over patients' wellbeing. In their interactions with pharmaceutical companies, private general practitioners (GPs) can experience the conflict of interest (COI), a situation whereby the impartiality of GPs' professional decision-making may be influenced by secondary interests such as financial gains from prescribing specific pharmaceutical brands.

Methods and analysis: This study is a randomised control trial (RCT) to assess the impact of a multifaceted intervention on GPs' medical practice. The study sample consists of 419 registered GPs who own/work in private clinics and will be randomly assigned to intervention and control groups. The intervention group GPs will be exposed to emotive and educational seminars on medical ethics, whereas control group GPs will be given seminars on general medical topics. The primary outcome measure will be GPs' prescribing practices, whereas the secondary outcome measures will be their knowledge and attitudes regarding COI that arises from pharmaceutical incentivisation. In addition to a novel standardised pharmaceutical representatives (SPSR) method, in which field researchers will simulate pharmaceutical marketing with GPs, pre-and post-surveys, and qualitative interviewing will be performed to collect data on GPs' knowledge, attitudes and practices in relation to COI linked with pharmaceutical incentives. Univariate and multivariate statistical analyses will be performed to measure a change in GPs' knowledge, attitudes, and practices, while qualitative analysis will add to our understanding of the quantitative SPSR data.

Ethics and dissemination: Ethics approval has been obtained from the Pakistan National Bioethics Committee (# 4-87/NBC-582/21/1364), the Aga Khan University (# 2020-4759-1129) and the London School of Hygiene and Tropical Medicine (# 26506). We will release results within 6 to 9 months of the study's completion.

Trial registration number: ISRCTN12294839

Keywords: protocols and guidelines, health policy, medical ethics, education and training, quality in healthcare, public health

Strengths and limitations of the study

1. The study assesses whether emotive sensitisation can help reduce profit-led prescribing.
2. It evaluates the potential usability of the SPSR method to ensure quality control.
3. It uses a mixed-method approach to perform a comprehensive assessment.
4. The inclusion of more female GPs would have provided additional insights.

For peer review only - http://bmjopen.bmj.com/site/about/guidelines.xhtml

Introduction

The pharmaceutical industry is often a vital source of information for general practitioners (GPs) to learn about pharmaceutical products. This information helps GPs make informed choices and decisions in relation to patients' diverse health needs [1]. Nevertheless, the relationship between the pharmaceutical industry and GPs can negatively affect patients' health and wellbeing, if/when incentives are mobilised to maximise profits through inappropriate prescribing [2]. As a result of pharmaceutical incentivisation, GPs may experience a conflict of interest (COI) - a situation where GPs' professional judgment is influenced by a secondary interest, such as financial gain, leading to a decision that conflicts with their interpretation of the patient's best interest [3, 4]. There is evidence that GPs who engage in pharmaceutical incentivisation are likely to prescribe unnecessary and/or more expensive medicines to patients, further contributing to an increased financial burden on them [5, 6]. In addition, pharmaceutical incentivisation has important implications for antimicrobial resistance (AMR), as to achieve pharmaceutical targets, GPs may prescribe antibiotics to patients with common self-resolving ailments like cold and flu [7].

In Pakistan, the health system is comprised of public and private sectors where a large proportion of patients access healthcare from private GPs [8]. At the same time, the country is home to over 600 pharmaceutical companies including multinational, national, and franchise-based local companies [9]. To compete in the market, pharmaceutical companies use incentivisation as a major tool to maximise profits from GPs' prescriptions [10]. On the other hand, in Pakistan, poverty represents one of the key problems that people and the inextricable link between poverty and a lack of education and health awareness gives perfect grounds to the

For peer review only - http://bmjopen.bmj.com/site/about/guidelines.xhtml

pharmaceutical industry and GPs to capitalise on patients' weaknesses to maximise profits from pharmaceutical products, further exacerbating problems for patients [11, 12].

Our previous research in Pakistan helped us identify three interlinked health system constraints that shape private GPs' decisions on receiving personal benefits from prescribing antibiotics [13-17]. First, insufficient income generation from patient consultations alone - as patients are unwilling to spend substantial sums on medical advice without a prescription - resulted in a reliance on other sources of income [16]. Second, limited opportunities for continuing medical education (CME) other than from pharmaceutical companies may lead to a close relationship with pharmaceutical sales representatives (PSRs) [14]. Third, inadequate access to diagnostic tools to establish the clinical need for antibiotics, making it easier for doctors to justify defensive over-prescribing [14]. We also found that private doctors engage in activities such as generating extra income from receiving monetary or non-monetary incentives, and sponsorships to attend academic events from pharmaceutical companies in return for meeting sales targets for specific medicines [14]. These behaviours of private physicians stem from deeply embedded dynamics of pluralistic health systems. Hence, not only did these studies provide rich insights into critical health system and policy dynamics, but also helped to identify the issue of COI in medical practice and how this contributes to unnecessary prescribing of medicines in primary care settings.

Therefore, to improve the quality of primary care, actions are required that address pharmaceutical incentivisation [18, 19]. However, major impediments to progress on this front include limited assessment of interventions for improving the quality of care delivered by private GPs [20, 21], and a marked absence of attention by researchers and policymakers as to the role of political, social, economic and cultural factors that influence the effectiveness and scalability

For peer review only - http://bmjopen.bmj.com/site/about/guidelines.xhtml

of interventions [22]. Furthermore, prohibiting or constraining private physicians through the enforcement of regulations may fail because of a lack of political and social support and enforcement capacity, as well as insufficient public sector capacity to provide care [20]. Training interventions focusing on increasing technical knowledge and skills are the most common approach used to improve the quality of care [23, 24]. Our previous research also suggests that an intervention that can enhance GPs' understanding of COI in medical practice might be useful to enable them to recognise potential actions that threaten professional ethics in their medical practice while interacting with PSRs [14, 16]. It is clear, however, from consistent evidence on the 'know-do' gap that COI related to profit generation from medicine sales plays a critical role in prescribing decisions, as do values associated with professional ethics and altruism [25, 26]. To improve GPs' understanding of COI in medical practice, we aim to develop an intervention based on emotive and educational seminars and to test their impact on private GPs' knowledge, attitudes, and practices in relation to professional ethics in medical practice.

Study objectives

The overarching aim of our study is to assess the impact of a multifaceted intervention for improving GPs' prescribing practices in relation to COI linked with pharmaceutical incentivisation. The specific objectives of the study are to:

- Alter GPs' prescribing practice in response to pharmaceutical incentives.
- Improve their knowledge of professional ethics in medical practice.
- Shift their attitudes to give primacy to patients' wellbeing rather than pharmaceutical incentives.

For peer review only - http://bmjopen.bmj.com/site/about/guidelines.xhtml

Methods

We plan to complete the study in three phases: formative, intervention, and evaluation. The study is being undertaken in Karachi which is the largest city by geographical area and population size in Pakistan [27]. In 2021, we completed the formative phase of the study, whereas the intervention and evaluation phases will be completed in 2022.

Formative work

We used semi-structured interviews to obtain information from GPs, policy actors, and PSRs about factors that lead to COI arising from pharmaceutical incentivisation. Individuals from regulatory institutions, professional medical associations, and health communication/media consultants were deemed policy actors. One of the key objectives of the formative work was to co-develop a multifaceted intervention that could improve GPs' knowledge, attitude, and practices about professional ethics in medical practice. Preliminary analysis of our formative work suggests that in Pakistan professional ethics in medical practice have gradually changed and engaging in pharmaceutical incentivisation has become a norm. Competition in the pharmaceutical market, GPs' desire to maximise income, combined with weaknesses in health regulation produce a context where an unethical relationship between the pharmaceutical industry and GPs is developed and sustained. Based on our findings, recommendations from the study participants, and existing research, a multifaceted intervention has been developed in which, GPs have been invited to seminars to get exposure to messages on medical ethics.

Intervention

Our intervention is based on multifaceted seminars. As indicated in figure 1, the intervention group has been given seminars on ethics in relation to COI linked with pharmaceutical

For peer review only - http://bmjopen.bmj.com/site/about/guidelines.xhtml

incentivisation, while the control group has received seminars on clinical issues such as thalassemia and anaemia. The intervention seminars have been developed in four major phases. First, international and national guidelines have been reviewed to develop a brief document on professional ethics in medical practice. The document covers several issues including pharmaceutical incentivisation in the form of money, other material/nonmaterial benefits, and the misuse of the medical license. The document has been endorsed by regulatory bodies at the federal and provincial levels including the Pakistan Medical Commission (PMC) and the Sindh Healthcare Commission (SHCC). Second, in collaboration with media/communication experts, a filmmaker has been hired to produce an emotive video highlighting the effects of profit-led prescribing on patients' wellbeing. Third, GPs have been invited to attend seminars on professional ethics in medical practice and on managing anaemia and thalassemia in primary care settings. The intervention group GPs has also been sent the PMC- and SHCC-endorsed brief on ethics in medical practice (reinforcement) and the shortened version of the emotive video (reminder).

The control seminars have been developed in three phases. First, we identify experts in thalassemia and anaemia research and take them on board to teach these topics to the control group GPs. Second, in collaboration with experts, we develop a short educational video on thalassemia and handouts on the management of thalassemia and anaemia in primary care settings. Third, we invite the control group GPs to attend the seminars.

Outcome measures

The primary outcome measure of the study is the number of GPs who refuse to take unethical pharmaceutical incentives from the standardised pharmaceutical sales representatives (SPSRs).

For peer review only - http://bmjopen.bmj.com/site/about/guidelines.xhtml

The secondary outcomes include GP's change in knowledge and attitudes about professional ethics in medical practice.

Participants

Private GPs, registered with the PMC and the SHCC are deemed eligible to participate in the study. GPs outside Karachi, those working in welfare clinics, dentists, consultants, and unqualified healthcare providers are excluded from the study.

Sample size

For the present study, a list of 1695 GPs, obtained from the SHCC has been considered a sample universe. From it, 419 GPs have been sampled using a systematic sampling technique. In our literature review, we identify no relevant surveys of private GPs in Pakistan, so we use data from two studies on medical students and trainee doctors, to calculate the sample size for our study. One survey of medical students shows that 81% favoured pharmaceutical sponsorship of events at medical colleges [28] and the other study on trainee doctors in public hospitals indicates that 57% did not know any code of medical ethics [29]. We estimate that 80% of private GPs in our sample will be classified as 'supporting pharmaceutical incentivisation' and assume that our intervention shifts this to 65%, in line with changes observed in the few interventional studies addressing COI in medical professionals [30, 31]. Using these assumptions, a sample size of between 130-135 per group gives a power of 80% to detect a difference owing to the intervention. As indicated in figure 2, the sample of 419 GPs was extracted in three steps. First, the entire list is screened to exclude 510 healthcare providers who do not meet our eligibility criteria. Second, information validation is performed by calling the remaining 1185 healthcare providers to verify important information such as clinic addresses, registration status, level of

For peer review only - http://bmjopen.bmj.com/site/about/guidelines.xhtml

education, and whether they are active/inactive in medical practice, further leading to the exclusion of 764 names. As part of a baseline survey on GPs' knowledge and attitudes about ethics in medical practice, 3 GPs have been further excluded from the sample who refuse to participate in the study.

Randomisation

The 419 GPs who completed the baseline survey and consented to attend the seminars, have been assigned to control and intervention groups, using a simple randomisation technique. Each GP in the list has been first assigned a unique identification number, following the sorting of the list with respect to six districts in Karachi, namely, East, West, Central, South, Korangi, and Malir. Each GP has then been assigned a random number using the excel spreadsheet. Third, within each district, GPs are assigned to control and intervention groups by sorting the list. The intervention and control groups comprise of 210 and 209 GPs, of which, 135 and 132 GPs have attended the seminars. To prevent bias, we have collaborated with a statistician outside the research team to perform the randomisation process. Participants and data collectors are blinded in relation to GPs' allocation to control and intervention groups.

Data collection

To measure the impact of the intervention, our data collection is underway, and we anticipate completing it in September/October 2022. We adopt a multi-method approach to collect data on GPs' knowledge, attitudes, and practices in relation to COI linked with pharmaceutical incentivisation. Pre- and post-intervention surveys will be used to assess a change in GPs' knowledge of professional medical ethics and attitudes about engaging in pharmaceutical incentivisation. To collect information about their practices with respect to taking incentives for

For peer review only - http://bmjopen.bmj.com/site/about/guidelines.xhtml

prescribing, a novel SPSR method will be used, in which trained field researchers will visit the GP clinics as pharmaceutical marketers to assess GPs' reactions to pharmaceutical incentivisation offers. The SPSRs will recite a script (prepared by the research team) to introduce themselves and the imaginary pharmaceutical company and will market antibiotics commonly prescribed. In this process, the SPSRs make a clear incentivisation offer to GPs for prescribing the medicines and assess whether GPs accept/reject the offer. Since the SPSRs will be representing new pharmaceutical companies and there will be less familiarity between them and GPs, it is anticipated that there can be GPs' responses, difficult to classify in terms of acceptance or rejection. Therefore, situations in which GPs ignore the SPSR offers or ask them to come back later will be classified as "undetermined". The SPSRs will complete an electronic questionnaire after each GP visit to record information such as GPs' demographic and clinical characteristics, and their responses to the incentivisation offer. To ensure the data quality, the research team will put several measures in place: recruiting field supervisors who will accompany the SPSRs to GP clinics and ensure the SPSRs' responses in the questionnaire reflect the interactions with GPs, WhatsApp voice notes to the research team to summarise the interactions and monitoring the SPSR data considering the voice notes as a reference. The questionnaire will be analysed by research team members (also blinded to the allocation of GPs), who will assign a binary outcome for each GP such as "refused incentives immediately", "refused incentives after being offered", "asked for and accepted an incentive", "offered and accepted an incentive", "undetermined/undecided". To prevent the SPSR assessment from being discovered as a simulation that not only can risk the intervention assessment but the SPSRs, in terms of their physical safety, each GP is paid a maximum of two visits. It is also important to note that the SPSRs are blinded to the allocation of GPs.

For peer review only - http://bmjopen.bmj.com/site/about/guidelines.xhtml

GPs have been made aware, through the consent procedure (supplementary file 1) in the baseline survey, that their behaviour will be assessed covertly at some stage over 6 months but will not know details of the assessment to avoid the Hawthorne effect. We considered this novel SPSR approach to be superior to a revealed preferences approach (e.g., based on medical records) because records of prescribing by private physicians usually do not exist or are of poor quality in Pakistan, and to a stated preference design as it would rely on honest reporting of behaviour that is highlighted as unethical in the COI presentation.

Finally, to explain the undecided/undetermined SPSR assessment outcomes, we will conduct 20-25 qualitative interviews with GPs and get information on the reasons why some GPs ignore talking to PSRs, asking them to come back later, and what it means when a GPs uses terms like "I am saturated/packed/engaged".

Data analysis

The primary outcome will be assessed by comparing the proportion of GPs classified as 'yes' to accepting an unethical benefit (according to the regulator's guidelines) for prescribing the SPSR's antibiotic between the control and intervention groups. The proportions will be compared using a two-proportion z-test. Secondary outcomes such as knowledge and attitude shifts will be assessed by comparing mean scores in survey responses of control and intervention groups using unpaired t-test and proportions scores in survey responses using the z-test.

Ethics and dissemination

Ethics approval has been obtained from the Pakistan National Bioethics Committee (# 4-87/NBC-582/21/1364), the Aga Khan University (# 2020-4759-1129) and the London School of

For peer review only

Hygiene and Tropical Medicine (# 26506). Key ethical guidelines about informed consent, voluntary participation, and maintaining participants' confidentiality will be observed throughout the research process.

Study results will be disseminated through open-access peer-reviewed publications targeting health promotion professionals, policymakers, and researchers. The study will also be presented at academic conferences in medicine and public health.

Patient and public involvement

None.

Discussion

Our study is the first to evaluate the impact of a multifaceted intervention on GPs' knowledge, attitudes, and practices in relation to COI linked with pharmaceutical incentives: an area that has not been well studied in Pakistan. It may guide the design of further studies and interventions to improve prescribing practices in private primary care settings. We have attempted to develop a comprehensive approach to analyse the issue of pharmaceutical incentivisation and to suggest potential ways to address it. Our main objective is to examine the relationship between the pharmaceutical industry and GPs and to develop and test an emotive seminar-based intervention to improve GPs' knowledge, attitudes, and practices about professional ethics in medical practice.

The strengths of the study include the fact that it systematically uses different levels of investigation including formative work to understand how GPs, policy actors, and PSRs conceptualise COI in medical practice, factors that contribute to pharmaceutical incentivisation, and the methods they deem important to reduce it. The study also includes collaborations with

For peer review only

important regulatory bodies such as the PMC and SHCC. These collaborations not only enable the smooth execution of this sensitive study but can further strengthen the impact of the intervention. The study involves a novel approach, including an emotive element to seminars-based intervention. In particular, the video on COI in medical practice as well as religious messages aims at a more impactful message for GPs towards recognition of the negative effect of pharmaceutical incentivisation on patients' wellbeing and health. Other creative methods include slogan competition and reinforcement of the message through shortened COI video reminders that aim to keep GPs engaged with messages on medical ethics delivered as part of the intervention. Finally, to the best of our knowledge, the use of SPSR methodology to evaluate GPs' practices regarding pharmaceutical incentivisation is novel. This assessment method has the potential to minimise bias in collecting information on how GPs react to incentivisation offers following exposure to the intervention on medical ethics.

The study should also be considered in terms of limitations. A smaller number of GPs in the seminars may ignore invitations due to conflicting professional and personal engagements. While this sample distribution is important to measure the impact of the seminars on GPs with similar characteristics (i.e., ethnic identities, sociocultural background, and financial status), it can potentially enable information contamination across the groups. The fact that in Pakistan the proportion of female physicians in private primary care clinics is low. As such the insights into the difference in knowledge, attitudes, and practices regarding medical ethics between male and female participants provided by our analysis may be limited. Finally, to limit the risk of SPSRs being identified as simulators, each SPSR will be limited to only 2 visits to each GP clinic.

We hope the study results will help us understand whether emotive seminars may be a useful way to address the issue of pharmaceutical incentivisation in Pakistan. The post-seminars

For peer review only - http://bmjopen.bmj.com/site/about/guidelines.xhtml

qualitative interviews with GP can in particular be of great importance in determining which aspects of ethics-related CME seminars need improvement or greater emphasis. Regulatory bodies including PMC and SHCC can potentially use these findings to introduce new programs for educating GPs on professional ethics in medical practice. Finally, the study can also potentially shape future interventions to help improve GPs' practices.

Data sharing plan

Owing to the potentially sensitive nature of our research on conflict of interest, and the legal or professional ramifications on doctors for contravening codes of ethical practice, data has been anonymised and some quotes that risk damaging the reputations of specific doctors or groups of doctors have been removed. This is in line with our commitments to both AKU's and LSHTM's ethics committees. Hence, all the dsata collected will be kept confidential and will stay with the AKU-LSHTM core team. Data can be made available upon reasonable requests, from the corresponding author.

Acknowledgements

We would like to acknowledge support from various people and institutions including Dr Robyna Irshad Khan and Dr Natasha Ali (Associate Deans at AKU), Dr Sameen Siddiqi (Chair, Community Health Sciences, AKU), the PMC, the SHCC, and the Centre for Biomedical Culture and Ethics (CBEC). We are also thankful to the staff at the AKU's microscopy laboratory to help us with managing the logistics of the seminars.

Authors' contributions

MK, SS, RH, MNN, and AmnaRS contributed to the study design. MNN contributed to

For peer review only

For peer review only - http://bmjopen.bmj.com/site/about/guidelines.xhtml

the drafting and writing of this protocol. MK, RH, SS, AfifahRS and AmnaRS contributed equally through critically reviewing and revising the proposal. AfifahRS developed the flowchart indicating the sampling and randomisation process. IA performed randomisation. SSK assisted in intervention design and delivery.

Funding statement

This work is supported by the UK Research and Innovation (UKRI) under grant number MR/T02349X/1.

Competing interests statement

The authors have no competing interests to declare.

References

1. Yager, J. and R.E. Feinstein, *Medical education meets pharma: moving ahead.* Academic Psychiatry, 2010. **34**(2): p. 92-97.
2. Sismondo, S., *Epistemic corruption, the pharmaceutical industry, and the body of medical science.* Frontiers in Research Metrics and Analytics, 2021. **6**: p. 614013.
3. Yousafzai, A., *Corruption in medical practice: Where do we stand?* 2015. **J Ayub Med Coll Abbottabad 2015;27(3)**.
4. Trap, B., *Practices of dispensing doctors-drug use and health economics*, in *International Conferences on Improving Use of Medicines*. 1997, Zimbabwe Essential Drugs Action Programme, Ministry of Health & Child Welfare.
5. Khazzaka, M., *Pharmaceutical marketing strategies' influence on physicians' prescribing pattern in Lebanon: ethics, gifts, and samples.* BMC health services research, 2019. **19**(1): p. 1-11.
6. Naqvi, A., et al., *Report: Interactions and conflicts of interests between prescribers and medical sales representatives (MSRs) regarding prescribing and drug promotion practices in Karachi, Pakistan.* Pakistan journal of pharmaceutical sciences, 2019. **32**(2): p. 687-695.
7. Morel, C.M., et al., *Industry incentives and antibiotic resistance: an introduction to the antibiotic susceptibility bonus.* The Journal of Antibiotics, 2020. **73**(7): p. 421-428.
8. Kurji, Z., Z.S. Premani, and Y. Mithani, *Analysis of the health care system of Pakistan: lessons learnt and way forward.* J Ayub Med Coll Abbottabad, 2016. **28**(3): p. 601.
9. Mehmood, K.K., F. Sonia, and A. Umar, *Impact of Organic Structure on Competitive Performance of Pharmaceutical Companies in Pakistan: Study of Mediating Roles.* Pakistan Journal of Social Sciences (PJSS), 2016. **36**(2).
10. Gul, R., et al., *Perceptions of and barriers to ethical promotion of pharmaceuticals in Pakistan: perspectives of medical representatives and doctors.* BMC medical ethics, 2021. **22**(1): p. 1-16.

For peer review only - http://bmjopen.bmj.com/site/about/guidelines.xhtml

11. Saleem, H., M.S. Shabbir, and B. Khan, *Re-examining multidimensional poverty in Pakistan: A new assessment of regional variations.* Global Business Review, 2021. **22**(6): p. 1441-1458.
12. Arsani, A.M., B. Ario, and A.F. Ramadhan, *Impact of education on poverty and health: Evidence from Indonesia.* Economics Development Analysis Journal, 2020. **9**(1): p. 87-96.
13. Aftab, W., et al., *Variations in regulations to control standards for training and licensing of physicians: a multi-country comparison.* Human resources for health, 2021. **19**(1): p. 1-10.
14. Khan, M.S., et al., *What are the barriers to implementing national antimicrobial resistance action plans? A novel mixed-methods policy analysis in Pakistan.* Health policy and planning, 2020. **35**(8): p. 973-982.
15. Khan, M.S., et al., *'LMICs as reservoirs of AMR': a comparative analysis of policy discourse on antimicrobial resistance with reference to Pakistan.* Health Policy and Planning, 2019. **34**(3): p. 178-187.
16. Khan, M., et al., *Should performance-based incentives be used to motivate health care providers? Views of health sector managers in Cambodia, China and Pakistan.* Health Economics, Policy and Law, 2020. **15**(2): p. 247-260.
17. Khan, M.S., et al., *How do external donors influence national health policy processes? Experiences of domestic policy actors in Cambodia and Pakistan.* Health policy and planning, 2018. **33**(2): p. 215-223.
18. Das, J. and J. Hammer, *Quality of Primary Care in Low-Income Countries: Facts and Economics.* Annual Review of Economics, 2014. **6**(1): p. 525-553.
19. Morgan, R., T. Ensor, and H. Waters, *Performance of private sector health care: implications for universal health coverage.* The Lancet, 2016. **388**(10044): p. 606-612.
20. Montagu, D. and C. Goodman, *Prohibit, constrain, encourage, or purchase: how should we engage with the private health-care sector?* Lancet, 2016. **388**(10044): p. 613-21.
21. Shah, N.M., W.R. Brieger, and D.H. Peters, *Can interventions improve health services from informal private providers in low and middle-income countries?: a comprehensive review of the literature.* Health Policy Plan, 2011. **26**(4): p. 275-87.
22. Ghaffar, A., et al., *Where is the policy in health policy and systems research agenda?* Bull World Health Organ, 2016. **94**(4): p. 306-8.
23. Chakraborty, S., S.A. D'Souza, and R.S. Northrup, *Improving private practitioner care of sick children: testing new approaches in rural Bihar.* Health Policy Plan, 2000. **15**(4): p. 400-7.
24. Marsh, V.M., et al., *Improving malaria home treatment by training drug retailers in rural Kenya.* Trop Med Int Health, 2004. **9**(4): p. 451-60.
25. Chuc, N.T., et al., *Management of childhood acute respiratory infections at private pharmacies in Vietnam.* Ann Pharmacother, 2001. **35**(10): p. 1283-8.
26. Leonard, K.L. and M.C. Masatu, *Professionalism and the know-do gap: exploring intrinsic motivation among health workers in Tanzania.* Health Economics, 2010. **19**(12): p. 1461-1477.
27. Yusuf, H., *Conflict dynamics in Karachi*. Vol. 19. 2012: US Institute of Peace Washington.
28. Siddiqui, U.T., et al., *Attitudes of medical students towards incentives offered by pharmaceutical companies-perspective from a developing nation-a cross sectional study.* BMC medical ethics, 2014. **15**(1): p. 1-10.
29. Imran, N., et al., *HEALTH ETHICS EDUCATION: KNOWLEDGE, ATTITUDES AND PRACTICE OF HEALTHCARE ETHICS AMONG INTERNS AND RESIDENTS IN PAKISTAN.* JPMI: Journal of Postgraduate Medical Institute, 2014. **28**(4).
30. Thirunavukarasu, P., et al., *Educational intervention is effective in improving knowledge and confidence in surgical ethics—a prospective study.* The American Journal of Surgery, 2010. **200**(5): p. 665-669.

For peer review only - http://bmjopen.bmj.com/site/about/guidelines.xhtml

31. Zakaria, A.M., W.F. Sleem, and A.M. Seada, *Effectiveness of ethical issues teaching program on knowledge, ethical behavior and ethical stress among nurses.* Journal of Nursing Education and Practice, 2016. **6**(7): p. 125.

Figure 1 legend: GP, general practitioner; SHCC, Sindh Healthcare Commission; PMC, Pakistan Medical Commission; COI, conflict of interest; SPSR, standardised pharmaceutical sales representative

Figure 1 caption: Diagram of the randomised control trial design

Figure 2 legend: GP, general practitioner; SHCC, Sindh Healthcare Commission.

Figure 2 caption: Flowchart of the sampling and randomisation process

For peer review only

For peer review only - http://bmjopen.bmj.com/site/about/guidelines.xhtml

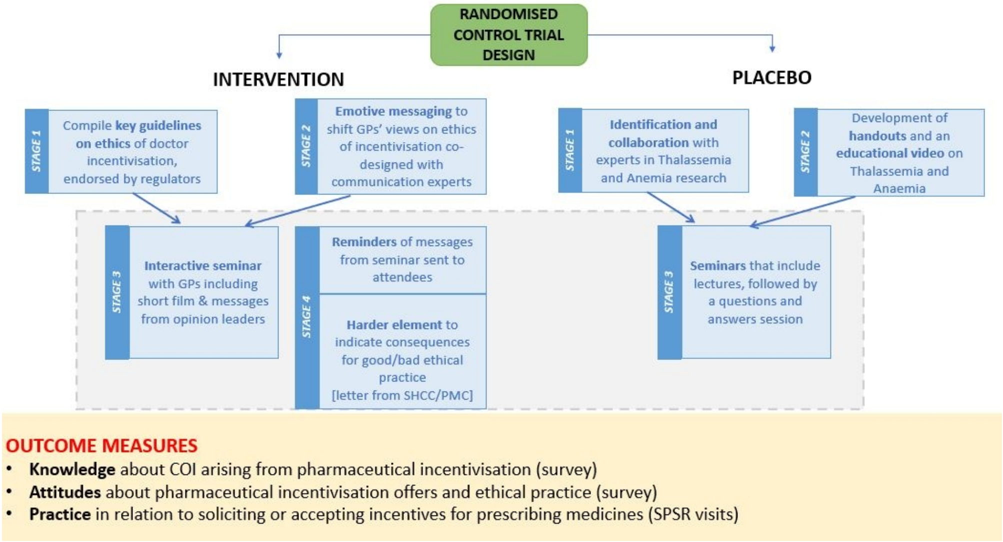

Figure 1: Diagram of the randomised control trial design

221x121mm (120 x 120 DPI)

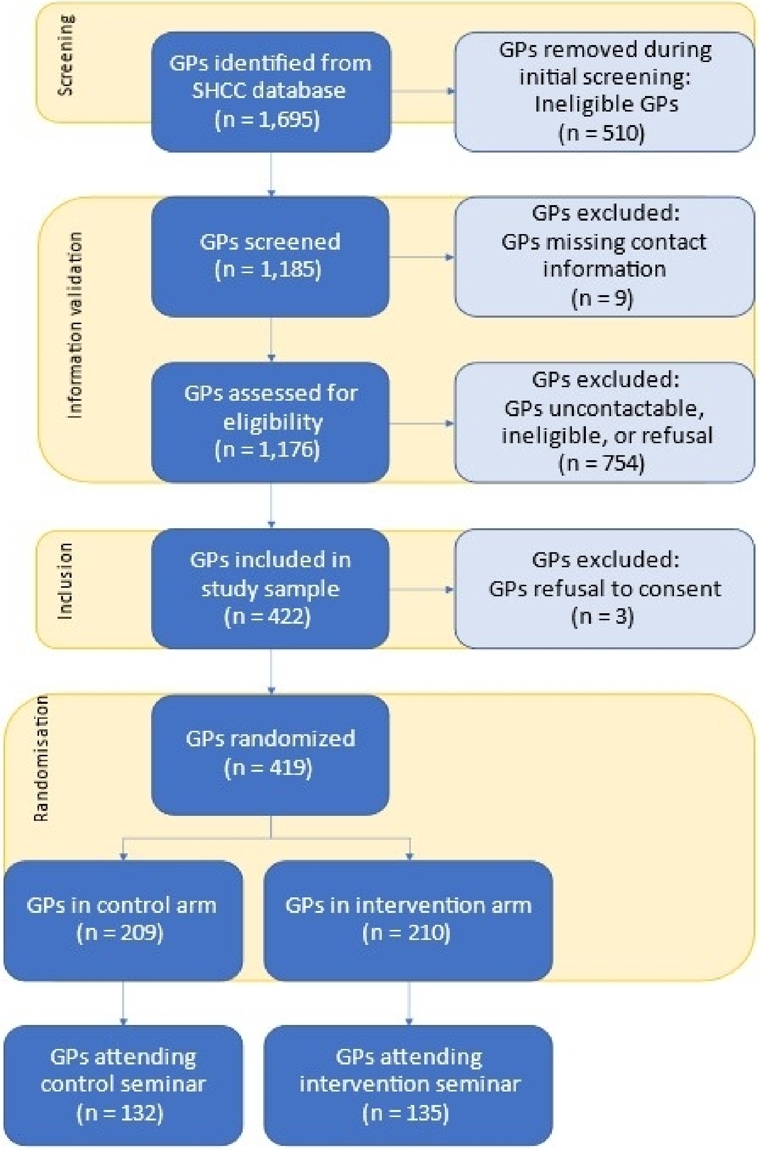

Figure 2: Flowchart of the sampling and randomisation process

125x186mm (96 x 96 DPI)

Supplementary File 1

PRESCRIBING PRACTICES IN PLURALISTIC HEALTH SYSTEMS IN PAKISTAN
Aga Khan University, Pakistan
Study ERC # 2021-6781-19734

Principal investigators: Dr. Sadia Shakoor, the Department of Pathology and Laboratory Medicine, and Dr Mishal Khan, London School of Hygiene and Tropical Medicine.

Co-Principal Investigators: Dr Rumina Hasan, Dr Naveed Noor, the Department of Pathology and Laboratory Medicine, Dr Sameen Siddiqui, and Dr Rehana Siddiqui, the Department of Community Health Sciences, Aga Khan University.

Institute: The Aga Khan University

For peer review only

INFORMATION SHEET

We would like to invite you to take part in a research study. Joining the study is entirely up to you. Before you decide, you need to understand why the research is being done and what it would involve. One of our team will go through this information sheet with you and answer any questions you may have.

Who is conducting this study?
We are a team of researchers from the London School of Hygiene and Tropical Medicine (LSHTM), and the Aga Khan University (AKU). The study is funded by the UK research councils and the sponsor is LSHTM.

This study has been reviewed and given favourable opinion by the LSHTM (UK) and AKU (Pakistan) ethics Committee (#1234 & # 2021-6781-19734).

Why are we conducting this research?
We are conducting a study that seeks to assess the impact of an education and motivation-based intervention on the knowledge, attitudes, and practice of doctors in Karachi, Pakistan. Findings will be valuable in Pakistan but also in other countries in Asia and around the world.

What will be done if I take part in this research?
If you choose to participate in this research, we will invite you to a seminar discussing important health-related issues. We are conducting an experimental study through seminars given to two groups of doctors. Each group will be taught different topics/contents related to health. After the seminars, we will explore which group learned more. The purpose of these seminars is to provide doctors with information that can improve their professional practice. You will be asked to complete a short survey before and after the seminar. You might also be selected to participate in a brief interview to give us your feedback on the seminar. Following the seminar, we may send you some reminders of the seminar content through your phone or at your clinic. Finally, within 6 months of the seminar, we will conduct an unannounced assessment through physical visits that will allow us to provide you feedback to enhance your professional practice. The assessment will also allow us to examine if our seminar was effective and to find ways to improve its delivery in the future.

For peer review only - http://bmjopen.bmj.com/site/about/guidelines.xhtml

Supplementary File 1

Why have I been selected?

In this study, the participants we are approaching are qualified doctors working as general practitioners (GPs) in Karachi. We are collaborating with the Sindh Healthcare Commission and have randomly selected approximately 300 doctors using a list of private clinics that they maintain.

How shall relevant audiences get to know the results of the study?

The research findings will be published in academic journals and presented at academic conferences and seminars (in Pakistan and internationally) so that doctors and other public health professionals can learn about the study findings. A report and presentation will be made to policy makers in Pakistan.

Are there any disadvantages or advantages involved in taking part?

There only individual benefits to participating is the opportunity to improve your knowledge and professional practice. Your participation may have wider benefits as it will help us to design materials or policies that might improve medical practice. Participating in this research should not cause any discomfort or hazards and you can stop participating at any time by letting a member of the research team know. Any feedback provided to you will be respectful and confidential.

Who will have access to the information I give?

We will not share individual information about you or other participants with anyone beyond our research team. Instead, the knowledge gained from this research will be shared in summary form (without revealing your name or personal details) with organizations or individuals that will find the information useful in improving policy.

We will ask for your consent specifically on whether

a) you are willing to participate in the research
b) whether you agree for us to enter the information you provide into a database that will allow it to be used for future research (without contacting you again)

How will my privacy and the confidentiality of my research records be protected?

Personal information will be used for introductory and familiarity purposes alone. Personal information will never be used in a publication or presentation. All information collected about you will be kept private, safe and secure. Only the study staff who check that the study is being carried out properly will be allowed to look at information about you. All your research data will be coded, meaning that each participant will only be identified with a number. Any data placed in a database or shared with other members of the research team will only contain information that cannot be traced back to you.

Your personal details, meaning your name and other identifiable information, will be kept in a different safe place to the other study information and will be destroyed within 10 years of the end of the study. At the end of the project, the study data will be archived on LSHTM's Secure Server. The data will be made available to other researchers worldwide for research and to improve medical knowledge and patient care. Your personal information will not be included and there is no way that you can be identified.

Where can I find out more about how my information is used?

- At https://www.lshtm.ac.uk/files/research-participant-privacy-notice.pdf
- by asking one of the research team
- by sending an email to DPO@lshtm.ac.uk

For peer review only - http://bmjopen.bmj.com/site/about/guidelines.xhtml

Supplementary File 1

Can I change my mind or refuse to participate?

Participation in this study is voluntary. You are free to decide if you want to take part or not. If you do agree, you can change your mind at any time. You can refuse to answer any specific questions or stop participating in the intervention at any time. If you choose not to answer a question, or even decide not to participate in the study at all, it will not affect you in any way today or in the future. If you stop being part of the study at any time, we will keep information about you that we already have.

What if I have any questions?

If you wish to receive further information on the research project, please do not hesitate to contact members of the research team, namely the following:

PI: Dr Sadia Shakoor, sadia.shakoor@aku.edu
CO-PI: Dr Rumina Hasan, rumina.hasan@aku.edu
CO-PI: Dr Naveed Noor, naveed.noor@aku.edu
+92 312 7275661

For peer review only

For peer review only - http://bmjopen.bmj.com/site/about/guidelines.xhtml

Supplementary File 1

CONSENT FORM

I hereby acknowledge that:

1. My signature, as witnessed by the Consent Taker, is my acknowledgement that I have agreed to participate in the interview.
2. I have received a copy of an information sheet that explains my role in this research. I understand its contents and agree to participate in this research.
3. I can withdraw from the research at any point of time
4. I consent / do not consent* to have the coded data made available for future research by putting it into a data repository
5. I agree / do not agree* to audio-recording of my participation in the research.
6. I agree/do not agree* for verbatim quotes to be used without identifying me.

*please circle as appropriate

This research has been explained to me in ____________________ (language) on ________ (date).

____

Name and Signature (Participant) Date

____

Name and Signature (Consent Taker) Date

For peer review only

STANDARD PROTOCOL ITEMS: RECOMMENDATIONS FOR INTERVENTIONAL TRIALS

SPIRIT 2013 Checklist: Recommended items to address in a clinical trial protocol and related documents*

Section/item	Item No	Description	Addressed on page number
**Administrative information**			
Title	1	Descriptive title identifying the study design, population, interventions, and, if applicable, trial acronym	2
Trial registration	2a	Trial identifier and registry name. If not yet registered, name of intended registry	2
	2b	All items from the World Health Organization Trial Registration Data Set	NA
Protocol version	3	Date and version identifier	NA
Funding	4	Sources and types of financial, material, and other support	15
Roles and responsibilities	5a	Names, affiliations, and roles of protocol contributors	1, 14-15
	5b	Name and contact information for the trial sponsor	15
	5c	Role of study sponsor and funders, if any, in study design; collection, management, analysis, and interpretation of data; writing of the report; and the decision to submit the report for publication, including whether they will have ultimate authority over any of these activities	NA
	5d	Composition, roles, and responsibilities of the coordinating centre, steering committee, endpoint adjudication committee, data management team, and other individuals or groups overseeing the trial, if applicable (see Item 21a for data monitoring committee)	15

For peer review only

**Introduction**			
Background and rationale	6a	Description of research question and justification for undertaking the trial, including summary of relevant studies (published and unpublished) examining benefits and harms for each intervention	3-5
	6b	Explanation for choice of comparators	NA
Objectives	7	Specific objectives or hypotheses	5
Trial design	8	Description of trial design including type of trial (eg, parallel group, crossover, factorial, single group), allocation ratio, and framework (eg, superiority, equivalence, noninferiority, exploratory)	7-9
**Methods: Participants, interventions, and outcomes**			
Study setting	9	Description of study settings (eg, community clinic, academic hospital) and list of countries where data will be collected. Reference to where list of study sites can be obtained	6
Eligibility criteria	10	Inclusion and exclusion criteria for participants. If applicable, eligibility criteria for study centres and individuals who will perform the interventions (eg, surgeons, psychotherapists)	8
Interventions	11a	Interventions for each group with sufficient detail to allow replication, including how and when they will be administered	7
	11b	Criteria for discontinuing or modifying allocated interventions for a given trial participant (eg, drug dose change in response to harms, participant request, or improving/worsening disease)	NA
	11c	Strategies to improve adherence to intervention protocols, and any procedures for monitoring adherence (eg, drug tablet return, laboratory tests)	NA
	11d	Relevant concomitant care and interventions that are permitted or prohibited during the trial	NA
Outcomes	12	Primary, secondary, and other outcomes, including the specific measurement variable (eg, systolic blood pressure), analysis metric (eg, change from baseline, final value, time to event), method of aggregation (eg, median, proportion), and time point for each outcome. Explanation of the clinical relevance of chosen efficacy and harm outcomes is strongly recommended	8<br>NA
Participant timeline	13	Time schedule of enrolment, interventions (including any run-ins and washouts), assessments, and visits for participants. A schematic diagram is highly recommended (see Figure)	8-9, figure 1: (randomisation)

For peer review only - http://bmjopen.bmj.com/site/about/guidelines.xhtml

Sample size	14	Estimated number of participants needed to achieve study objectives and how it was determined, including clinical and statistical assumptions supporting any sample size calculations	8
Recruitment	15	Strategies for achieving adequate participant enrolment to reach target sample size	NA

Methods: Assignment of interventions (for controlled trials)

Allocation:

Sequence generation	16a	Method of generating the allocation sequence (eg, computer-generated random numbers), and list of any factors for stratification. To reduce predictability of a random sequence, details of any planned restriction (eg, blocking) should be provided in a separate document that is unavailable to those who enrol participants or assign interventions	9
Allocation concealment mechanism	16b	Mechanism of implementing the allocation sequence (eg, central telephone; sequentially numbered, opaque, sealed envelopes), describing any steps to conceal the sequence until interventions are assigned	9
Implementation	16c	Who will generate the allocation sequence, who will enrol participants, and who will assign participants to interventions	9
Blinding (masking)	17a	Who will be blinded after assignment to interventions (eg, trial participants, care providers, outcome assessors, data analysts), and how	9
	17b	If blinded, circumstances under which unblinding is permissible, and procedure for revealing a participant's allocated intervention during the trial	NA

Methods: Data collection, management, and analysis

Data collection methods	18a	Plans for assessment and collection of outcome, baseline, and other trial data, including any related processes to promote data quality (eg, duplicate measurements, training of assessors) and a description of study instruments (eg, questionnaires, laboratory tests) along with their reliability and validity, if known. Reference to where data collection forms can be found, if not in the protocol	9-11
	18b	Plans to promote participant retention and complete follow-up, including list of any outcome data to be collected for participants who discontinue or deviate from intervention protocols	NA

Data management	19	Plans for data entry, coding, security, and storage, including any related processes to promote data quality (eg, double data entry; range checks for data values). Reference to where details of data management procedures can be found, if not in the protocol	10
Statistical methods	20a	Statistical methods for analysing primary and secondary outcomes. Reference to where other details of the statistical analysis plan can be found, if not in the protocol	11
	20b	Methods for any additional analyses (eg, subgroup and adjusted analyses)	NA
	20c	Definition of analysis population relating to protocol non-adherence (eg, as randomised analysis), and any statistical methods to handle missing data (eg, multiple imputation)	NA
**Methods: Monitoring**			
Data monitoring	21a	Composition of data monitoring committee (DMC); summary of its role and reporting structure; statement of whether it is independent from the sponsor and competing interests; and reference to where further details about its charter can be found, if not in the protocol. Alternatively, an explanation of why a DMC is not needed	NA
	21b	Description of any interim analyses and stopping guidelines, including who will have access to these interim results and make the final decision to terminate the trial	NA
Harms	22	Plans for collecting, assessing, reporting, and managing solicited and spontaneously reported adverse events and other unintended effects of trial interventions or trial conduct	NA
Auditing	23	Frequency and procedures for auditing trial conduct, if any, and whether the process will be independent from investigators and the sponsor	NA
**Ethics and dissemination**			
Research ethics approval	24	Plans for seeking research ethics committee/institutional review board (REC/IRB) approval	11-12
Protocol amendments	25	Plans for communicating important protocol modifications (eg, changes to eligibility criteria, outcomes, analyses) to relevant parties (eg, investigators, REC/IRBs, trial participants, trial registries, journals, regulators)	NA

For peer review only - http://bmjopen.bmj.com/site/about/guidelines.xhtml

Consent or assent	26a	Who will obtain informed consent or assent from potential trial participants or authorised surrogates, and how (see Item 32)	9, 11-12
	26b	Additional consent provisions for collection and use of participant data and biological specimens in ancillary studies, if applicable	NA
Confidentiality	27	How personal information about potential and enrolled participants will be collected, shared, and maintained in order to protect confidentiality before, during, and after the trial	12
Declaration of interests	28	Financial and other competing interests for principal investigators for the overall trial and each study site	15
Access to data	29	Statement of who will have access to the final trial dataset, and disclosure of contractual agreements that limit such access for investigators	14
Ancillary and post-trial care	30	Provisions, if any, for ancillary and post-trial care, and for compensation to those who suffer harm from trial participation	NA
Dissemination policy	31a	Plans for investigators and sponsor to communicate trial results to participants, healthcare professionals, the public, and other relevant groups (eg, via publication, reporting in results databases, or other data sharing arrangements), including any publication restrictions	11-12
	31b	Authorship eligibility guidelines and any intended use of professional writers	NA
	31c	Plans, if any, for granting public access to the full protocol, participant-level dataset, and statistical code	14
**Appendices**			
Informed consent materials	32	Model consent form and other related documentation given to participants and authorised surrogates	Appendix 1
Biological specimens	33	Plans for collection, laboratory evaluation, and storage of biological specimens for genetic or molecular analysis in the current trial and for future use in ancillary studies, if applicable	NA

*It is strongly recommended that this checklist be read in conjunction with the SPIRIT 2013 Explanation & Elaboration for important clarification on the items. Amendments to the protocol should be tracked and dated. The SPIRIT checklist is copyrighted by the SPIRIT Group under the Creative Commons "Attribution-NonCommercial-NoDerivs 3.0 Unported" license.

For peer review only - http://bmjopen.bmj.com/site/about/guidelines.xhtml
