# Supplementary material for: Impact of a multifaceted intervention on physicians’ knowledge, attitudes and practices in relation to pharmaceutical incentivisation: protocol for a randomised control trial
Source: BMJ Open. 2022 Nov 4;12(11):e067233. doi: 10.1136/bmjopen-2022-067233 (PMC9639112; doi:10.1136/bmjopen-2022-067233)
Supplement: Reviewer comments [file bmjopen-2022-067233.reviewer_comments.pdf]

PEER REVIEW HISTORY

BMJ Open publishes all reviews undertaken for accepted manuscripts. Reviewers are asked to complete a checklist review form (**http://bmjopen.bmj.com/site/about/resources/checklist.pdf**) and are provided with free text boxes to elaborate on their assessment. These free text comments are reproduced below.

ARTICLE DETAILS

**TITLE (PROVISIONAL)**	The impact of a multifaceted intervention on physicians' knowledge, attitudes, and practices in relation to pharmaceutical incentivisation: protocol for a randomised control trial
**AUTHORS**	Noor, Muhammad Naveed; Khan, Mishal; Rahman-Shepherd, Afifah; Siddiqui, Amna Rehana; Khan, Sabeen; Azam, Iqbal; Shakoor, Sadia; Hasan, Rumina

This article was not externally reviewed at BMJ Open. Protocols that have been independently assessed prior to submission to BMJ Open are usually fast-tracked to publication on the grounds that further substantial changes will not be possible. This independent assessment will usually be external, independent review for both a substantial grant award from a non-commercial or government funder and ethics approval.
